# Adaptive alien genes are maintained amid a vanishing introgression footprint in a sea squirt

**DOI:** 10.1093/evlett/qrae016

**Published:** 2024-04-27

**Authors:** Fanny Touchard, Frédérique Cerqueira, Nicolas Bierne, Frédérique Viard

**Affiliations:** ISEM, University of Montpellier, CNRS, EPHE, IRD, Montpellier, France; ISEM, University of Montpellier, CNRS, EPHE, IRD, Montpellier, France; ISEM, University of Montpellier, CNRS, EPHE, IRD, Montpellier, France; ISEM, University of Montpellier, CNRS, EPHE, IRD, Montpellier, France

**Keywords:** biological invasions, adaptive introgression, selective sweep, local adaptation, anthropogenic hybridization, marinas

## Abstract

Human transport of species across oceans disrupts natural dispersal barriers and facilitates hybridization between previously allopatric species. The recent introduction of the North Pacific sea squirt, *Ciona robusta*, into the native range of the North Atlantic sea squirt, *Ciona intestinalis*, is a good example of this outcome. Recent studies have revealed an adaptive introgression in a single chromosomal region from the introduced into the native species. Here, we monitored this adaptive introgression over time, examining both the frequency of adaptive alleles at the core and the hitchhiking footprint in the shoulders of the introgression island by studying a thousand *Ciona* spp. individuals collected in 22 ports of the contact zone, 14 of which were sampled 20 generations apart. For that purpose, we developed a KASP multiplex genotyping approach, which proved effective in identifying native, nonindigenous and hybrid individuals and in detecting introgressed haplotypes. We found no early generation hybrids in the entire sample, and field observations suggest a decline in the introduced species. At the core region of the introgression sweep, where the frequency of *C. robusta* alleles is the highest and local adaptation genes must be, we observed stable frequencies of adaptive alien alleles in both space and time. In contrast, we observed erosion of *C. robusta* ancestry tracts in flanking chromosomal shoulders on the edges of the core, consistent with the second phase of a local sweep and a purge of hitchhiked incompatible mutations. We hypothesize that adaptive introgression may have modified the competition relationships between the native and invasive species in human-altered environments.

## Introduction

Human activities are responsible for ever-increasing biological introductions (Seebens et al., 2017), and the marine environment is no exception (Bailey et al., 2020; Sardain et al., 2019). Anthropogenic transport of species facilitates secondary contact between previously isolated species or lineages by altering connectivity pathways and acting as corridors and stepping stones (Alter et al., 2021; Bishop et al., 2017). These secondary contacts can result in anthropogenic hybridizations (i.e., human-mediated hybridizations; McFarlane & Pemberton, 2019) between native and nonindigenous species or lineages. They represent evolutionary experiments at the human scale for the study of the mechanisms and consequences of hybridization (Viard et al., 2020).

One particularly interesting outcome of anthropogenic hybridization is adaptive introgression. This process occurs when the incorporation of beneficial alleles through introgressive hybridization leads to an increased frequency of these alleles in the recipient populations (Hedrick, 2013). Cases of adaptive introgression have been reported where specific introgressed genomic regions are under selection in association with adaptive traits such as pollution or pesticide resistance (Oziolor et al., 2019; Suarez-Gonzalez et al., 2016; Valencia-Montoya et al., 2020; Le Corre et al., 2020). Adaptive introgression can be viewed as a form of selection on standing genetic variation that allows for rapid adaptation to changing environmental conditions (Norris et al., 2015; Oziolor et al., 2019). Examining changes in the frequency and the spatial distribution of introgressed alleles over time is particularly helpful to understanding the selection processes at play (e.g., local adaptation vs. strict positive selection) and the relative role played by selection and gene flow in maintaining locally adaptive genes. To date, however, there has been little monitoring of adaptive introgression over time (but see Norris et al. (2015) for a detailed study of pesticide resistance introgression in mosquitoes).

Anthropogenic hybridization between two tunicate species of the genus *Ciona*, following the introduction of *Ciona robusta* in the European native range of *Ciona intestinalis* in the early 2000s (Bouchemousse et al., 2016a), has resulted in an adaptive introgression island (Fraïsse et al., 2022). The two tunicate species are found in syntopy, with overlapping life cycles, in ports of the English Channel and the North-East Atlantic Ocean (Bouchemousse et al., 2016a,b). Moreover, F1-hybrids are successfully obtained in the laboratory at high rates but in one direction only, with sperm from *C. robusta* and eggs of *C. intestinalis* (Bouchemousse et al., 2016b; Malfant et al., 2018). Hybridization is extremely rare in natural populations, though (Bouchemousse et al., 2016b,c). For example, less than 0.03% of F1-hybrids (over 3,048 individuals) were reported by Bouchemousse et al. (2016b). However, introgression by *C. robusta* alleles was found in *C. intestinalis* populations sampled in 2012 in the European contact zone (Le Moan et al., 2021). Introgression is mostly found in a unique and localized genomic region between positions 700 kb and 1.5 Mb of chromosome 5 and is characterized by long tracts (30–150 kb) of *C. robusta* ancestry (Fraïsse et al., 2022). This introgression event is recent (estimated about 75 years ago) and not observed in populations outside the contact zone (Fraïsse et al., 2022; Le Moan et al., 2021). Although the introgressed haplotypes are not fixed in any study population, several pieces of evidence point toward adaptive introgression. Fraïsse et al. (2022) found that positive selection on the chromosome 5 introgression island is supported by long-range linkage disequilibrium patterns, haplotype-based neutrality tests and the presence of a tandem repeat of a cytochrome P450 gene in the core of the introgression island. This tandem repeat is a likely candidate for selection, possibly linked to detoxification functions often associated with cytochrome P450 genes (Fraïsse et al., 2022).

To get further insights into how selective processes shape introgression patterns, we examined the spatiotemporal dynamics of introgressed *C. robusta* allele frequencies. We analyzed 1,214 *Ciona* individuals collected during two time periods, spanning about 20 generations, in the European contact zone from the North Sea to the Bay of Biscay. Taking advantage of previous genomic resources (Le Moan et al., 2021; Fraïsse et al., 2022) and published genomes (Satou et al., 2019), we developed a KASP genotyping approach based on 23 ancestry-informative SNP markers specifically designed to distinguish native, nonnative and hybrid individuals as well as to identify alien alleles across the introgression island on chromosome 5. We first confirmed ongoing hybridization between the two species is very rare and updated the spatial distribution of *C. robusta* alleles. Our study also exemplified the benefits of spatiotemporal analyses of adaptive introgression. While we observed stability in both time and space of the frequency of *C. robusta* alleles at the core of the introgression island, we detected decreasing frequencies in the flanking regions (“shoulders” *sensu*Schrider et al., 2015) in most populations and a tendency for weaker linkage disequilibria. Altogether, the observed pattern suggests a kind of balancing selection (e.g., local adaptation) at the adaptive locus amid counter-selection of alien hitchhiker alleles in the native genome or their replacement by native alleles through gene flow (i.e., the second phase of a local sweep; Bierne, 2010).

## Methods

Additional information is provided in the Supplementary Material (section Supplementary Methods).

### Sampling and DNA extraction

**Figure 1:**
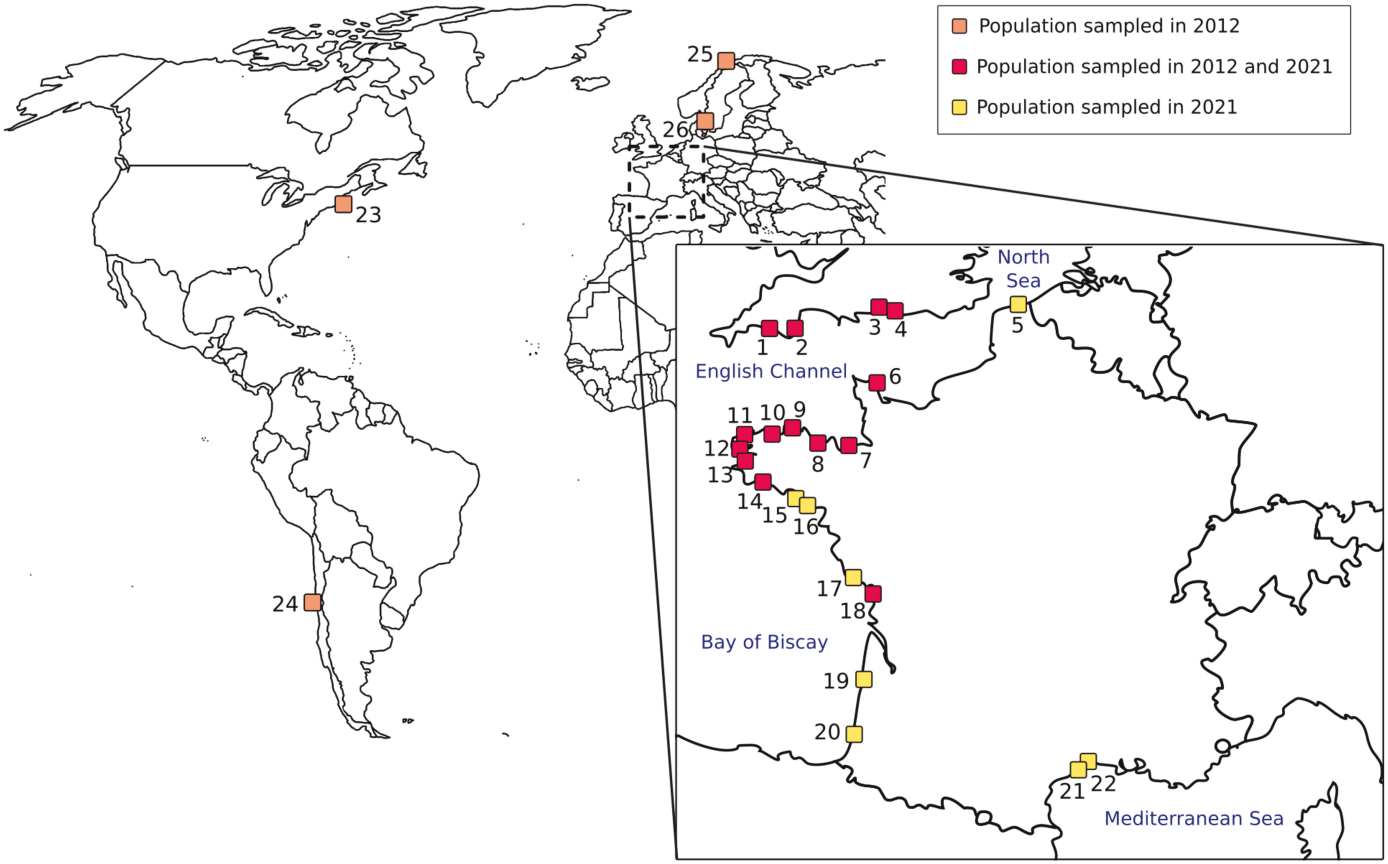
Sampling locations of *Ciona* spp. populations, in the European contact zone along the coasts of France and the United Kingdom (yellow (no.5, 15–17, 19–22) and red squares (no.1–4, 6–14, 18)) and outside the contact zone (orange squares (no.23–26)). Locations associated with the numbers on the map can be found in Supplementary Table S1.

*Ciona intestinalis* and *C. robusta* adult individuals were sampled in 22 ports from the North Sea to the Western Mediterranean Sea during late summer 2021, except for one locality (Dunkerque, site 5) sampled during summer 2022, totalizing 775 individuals (Figure 1; Supplementary Table S1) (time period referred as 2021 hereafter). In addition, for temporal analyses, DNAs of 439 *C. intestinalis* individuals, collected in the summer of 2011, 2012, and 2014 in 14 English and French ports also sampled in 2021, were included (time period referred to as 2012 hereafter; Supplementary Table S1). DNAs obtained from samples collected outside the contact zone as well as nine F1-hybrids were also included to be used as controls for our genotyping procedure. More details about sampling and DNA extractions are provided in the Supplementary Material.

### KASP genotyping

**Figure 2:**
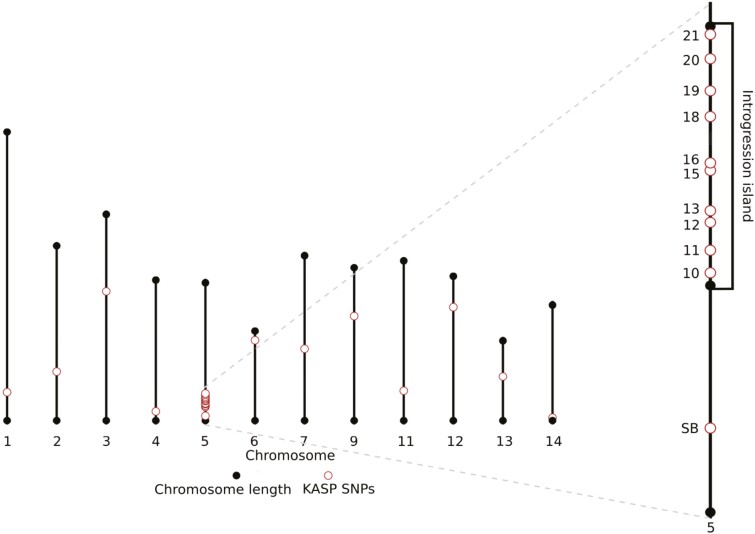
Position of the 23 nuclear SNPs retained for routine genotyping along the chromosomes of *C. intestinalis*, with a focus on the introgression island on chromosome 5.

Following a testing procedure (detailed in Supplementary Material), a total of 23 SNPs were analyzed for routine KASP genotyping. They include (Figure 2; Supplementary Table S3) (i) one SNP located on the mitochondrial genome, (ii) 11 SNPs along chromosome 5 (10 inside the introgression island and one SNP “SB,” outside (Figure 2)), and (iii) one SNP on each remaining chromosome (except chromosome 8 and 10), thus a further 11 SNPs that were pooled in one multiplex to estimate a hybrid index for the remainder of the genome, as in Hammel et al. (2024). Regarding chromosome 5, using genome sequences from Fraïsse et al. (2022) and the ddRADseq dataset from Le Moan et al. (2021), we identified one SNP shared by the two datasets (SNP 15 in Figure 2; SNP 38 radtag 877292-877271 in ddRADseq). We used this SNP as a marker of introgression frequency as it is located at the very core of the introgression island, 311 base pairs (bp) away from the candidate cytochrome P450 tandem repeat. The other 10 markers were used to study the extent of hitchhiking on the chromosome.

For genotyping, an end-point PCR was done (Supplementary Table S2) using 1 µl of assay mix (KASP-TF V4.0 2× Master Mix, 1×; primers, 1 µM; HyClone HyPure water) and 0.5 µl of DNA were mixed in qPCR 384-well plates using Labcyte Echo525. Four samples (one *C. robusta*, one nonintrogressed *C. intestinalis* and two introgressed *C. intestinalis)* were used as positive controls in every qPCR run. For each DNA and assay (12 simplexes and 1 multiplex), 2 fluorescence values were measured during the reading step, each associated with a given diagnostic allele. The fluorescence data were then analyzed using the variant allele fluorescent fraction (VAFF), which is calculated $\frac{f1}{f1+f2}$ with fi the fluorescence specific to the i allele (Hammel et al., 2024). The VAFF value indicates if, at a given SNP, an individual is homozygous (high or low value depending on the allele) or heterozygous (intermediate value), or it provides an estimate of the hybrid index when multiplexed (see Supplementary Methods).

### Data analysis

All analyses were performed in R (v4.2.2). Based on the VAFF value and the positive controls, genotypes were manually assigned to each sample for each SNP by determining cutoffs for each run (Supplementary Figure S1; Supplementary Table S4). All genotypes for the introgression island are found in Supplementary Figure S2, which visualizes the introgression tracts. The multiplex assay was used to compute the hybrid index and assign each individual to three categories: pure *C. robusta*, pure *C. intestinalis* or admixed individuals.

The next analyses were performed on a dataset consisting of only *C. intestinalis* individuals, both introgressed and nonintrogressed. We first examined SNP 15, at the core of the introgression island, and computed the *C. robusta* allele counts, genotype counts and allele frequency for the two sampling periods. The temporal variation of alien allele and genotype counts was analyzed over the whole dataset and per population by performing an exact test (Raymond & Rousset, 1995) using the *test-diff* function of the genepop v 1.1.7 R library. To account for multiple testing, we adjusted *p*-values using the Benjamini–Hochberg method with the *p.adjust* function in R. For each SNP located in the flanking regions of SNP 15 along chromosome 5, the Fisher’s exact test was used over the whole dataset. In addition, to examine the directionality of change over time, variations of allele frequencies between 2012 and 2021 were also analyzed using a one-sided Wilcoxon signed-rank test using populations as replicates. Finally, linkage disequilibria were estimated by the correlation coefficient *r*² of allele frequencies between SNP pairs using PLINK (v1.90b6.26) in both sampling periods for populations with a frequency of *C. robusta* allele at the core SNP 15 higher than 0.05 (diversity was too low in other population samples).

## Results

### Genotyping accuracy and verification of species status

Across the 1,291 analyzed individuals, only two were removed because of bad amplifications. The mitochondrial SNP and the multiplex confirmed the species identification that was done in the field (concordant for all except one individual). For the mitochondrial SNP, this result is consistent with previous studies (Bouchemousse et al., 2016b). The multiplex did not reveal any individual with intermediate VAFF values, indicating the absence of early generation hybrids in the contact zone for both time periods. None of the 109 *C. robusta* individuals displayed evidence of admixture. Outside the contact zone, two *C. intestinalis* individuals from Norway (site 25) were heterozygous at SNP 13; otherwise, no individual was introgressed or admixed in any of the four locations. As Norwegian *C. intestinalis* are outside the contact zone and not introgressed, this is likely due to shared polymorphism between *C. intestinalis* and *C. robusta* at SNP 13.

### Stable frequencies at SNP 15, the core of the introgression island

As expected, *C. robusta* allele frequency was the highest at SNP 15 both in 2012 and 2021, with an average value of 0.28 and 0.25, respectively. Allelic distribution was not different between the two time periods (Fisher’s exact test, *p* = 0.49), and neither was genotypic distribution (Fisher’s exact test, *p* = 0.55).

**Figure 3:**
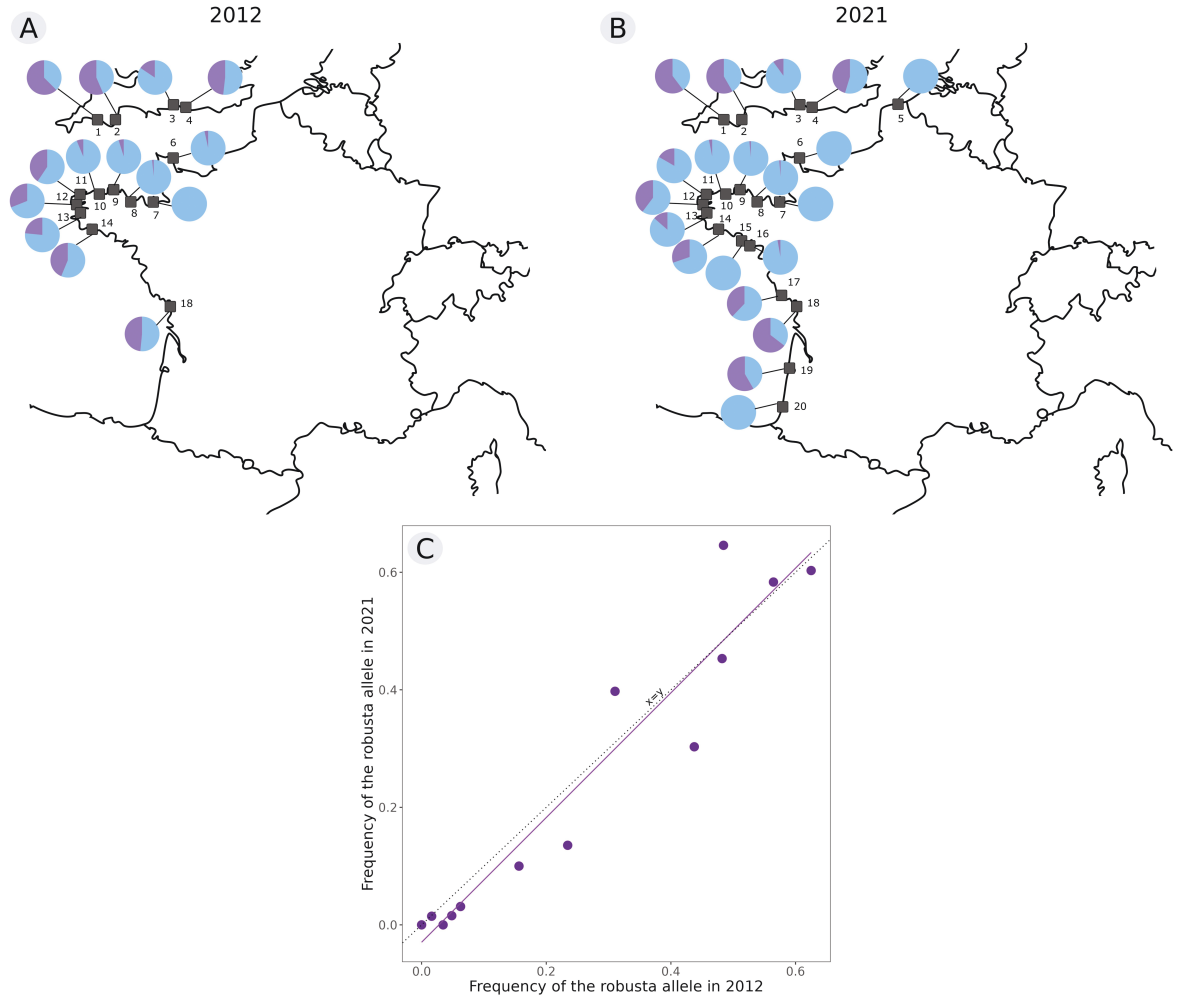
Distribution of introgressed *Ciona robusta* alleles over time. The maps show the relative proportion of introgressed *C. robusta* alleles (in purple on the pie charts) and *C. intestinalis* alleles (in blue) at core SNP 15 in 2012 (A) and in 2021 (B) in each study population. In (C), the frequency of *C. robusta* introgressed alleles for each population in 2012 (*x*-axis) and 2021 (*y*-axis) is shown compared to the 1:1 regression line (dotted line).

The *C. robusta* allele at SNP 15 was detected in 13 of the 14 populations sampled in 2012 and in 15 out of the 20 populations sampled in 2021 (Figure 3). In St-Vaast (site 6), the alien allele was detected in 2012 but at very low frequency (0.034) and no longer detected in 2021. At Hossegor (site 20), only one (nonintrogressed) individual of *C. intestinalis* could be found in the field. This sample was, therefore, not included in further analyses. Introgression was detected in three of the five other newly sampled populations (Trinité-sur-Mer, Bourgenay and Arcachon; sites 16, 17, and 19, respectively). When analyzing each population separately and adjusting for multiple testing, there was no significant difference in the alien allele frequency through time (Supplementary Table S5A).

Over the whole sampling zone, we can distinguish three areas where there is a high *C. robusta* allele frequency at SNP 15 in the populations at both time periods (Figure 3): South England (sites 1–4), West Brittany (sites 11–14) as well as around the Gironde estuary (sites 17–19), with an average *C. robusta* allele frequency of 0.44, 0.26, and 0.55, respectively.

### Temporal erosion in *C. robusta* ancestry on the shoulders of the introgression island

**Figure 4:**
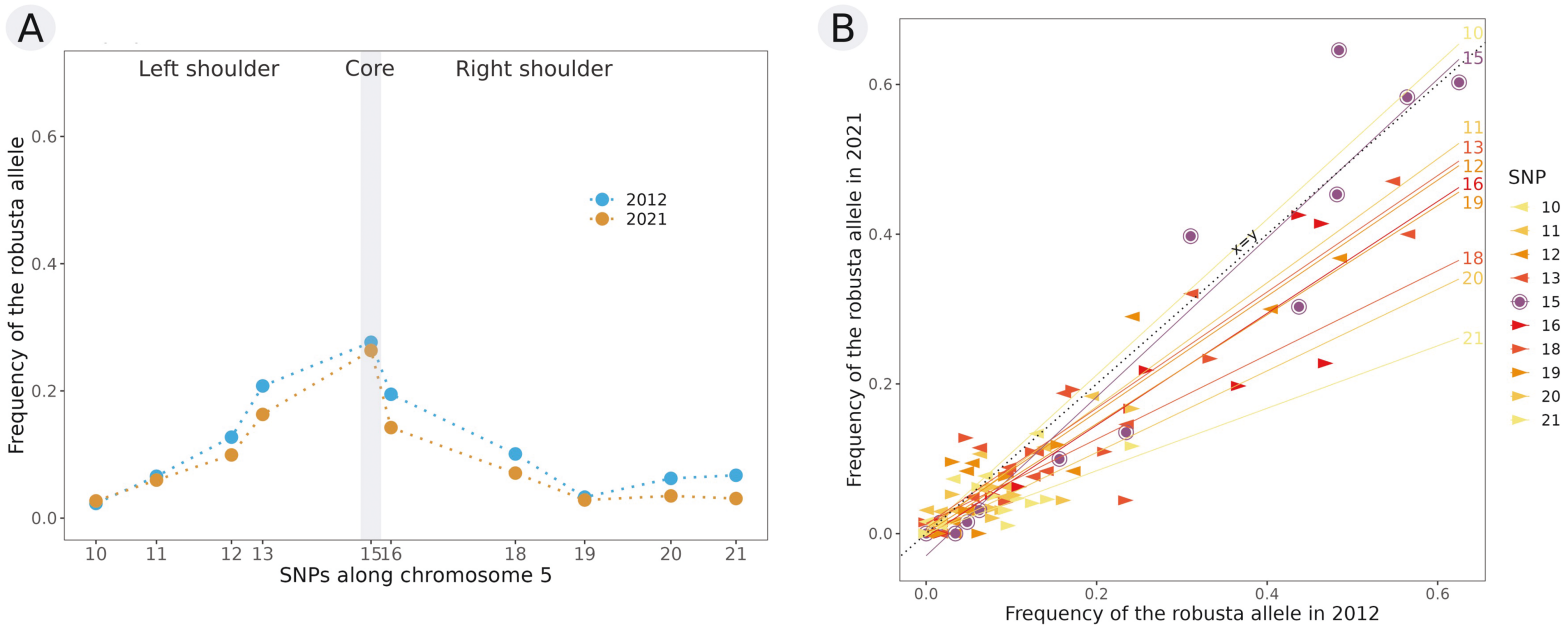
Comparison over time of the mean *Ciona robusta* allele frequency for each SNP in the introgression island of chromosome 5: (A) overall individuals (*N*_2012_ = 434; *N*_2021_ = 664) in 2012 (blue) and in 2021 (orange) (see Supplementary Figure S3 for graphs per population), and (B) frequencies for each SNP in each population in 2012 (*x*-axis) and 2021 (*y*-axis) compared to the 1:1 regression line (plain black line).

Following the analysis of allele frequencies at SNP 15, we then examined allele frequencies at SNPs flanking the core region in the shoulders of the introgression island. The *C. robusta* allele frequency decreases with the distance from SNP 15 as expected, although a slight rebound is observed at SNPs 20 and 21 in 2012 (Figure 4 and Supplementary Figure S3).

In contrast to the results for SNP 15, we observed a general decrease of the *C. robusta* allele frequency at every SNP located on the shoulders of the introgression island (Figure 4 and Supplementary Figure S4). Six SNPs (SNPs 12, 13, 16, 18, 20, and 21) showed significant changes in *C. robusta* allele frequencies (Fisher’s exact test in Supplementary Table S5B). In addition, five SNPs (13, 16, 18, 20, and 21) showed a significantly lower alien allele frequency in 2021 as compared with 2012 (Wilcoxon signed-rank test; Supplementary Table S5B). The SNPs 20 and 21 rebound observed in 2012 has been flattened out to lower frequencies in 2021.

**Figure 5:**
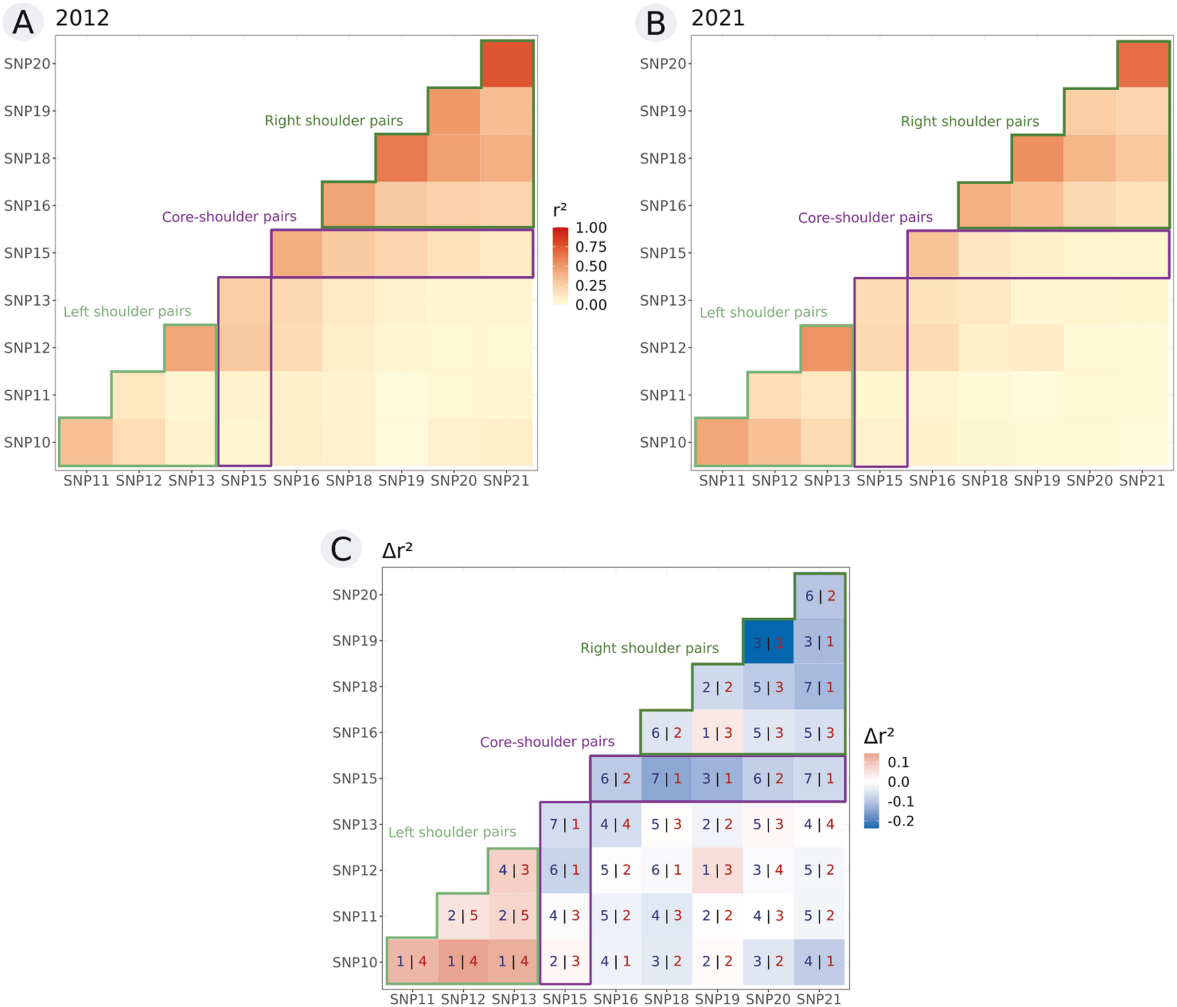
Linkage disequilibrium measured by correlation coefficient *r*² between each pair of SNPs for both 2012 (A) and 2021 (B). Cells are coloured based on the values; the higher the coefficient is, the darker the cell. *r*² values are provided in Supplementary Table S6. (C) represents the difference in *r*² values between the two years for each pair of SNPs, blue indicating a decrease in association from 2012 to 2021, red an increase and white no difference (the number of populations showing a decrease versus an increase is indicated with the same color code within each cell). In each panel, SNP pairs involved in the right and left shoulders (green line) and SNP pairs involving SNP15 (i.e., the core; violet line) are highlighted.

Because recombination breaks associations between SNPs over time, we expected to observe a decrease in linkage disequilibrium along the introgression region, notably between the core and shoulder SNPs. The mean values of *r*² measuring linkage disequilibrium were 0.176 in 2012 and 0.122 in 2021 (values per SNP pairs provided in Supplementary Table S6). The most associated loci were SNPs 20 and 21, SNPs 15 and 16, and SNPs 12 and 13 (Figure 5A and B). The last two pairs are also the ones with the smallest physical distances apart on the chromosome (respectively, around 22 kb and 35 kb); meanwhile, SNPs 20 and 21 are 74Kb apart. By comparing pairwise values between years, we did indeed observe a decrease in *r*² over time for most pairs (32 out of 45, Figure 5). Given the nonindependence between SNP pairs, we analyzed separately pairs between core SNP 15 and shoulder SNPs (both sides), left shoulder SNP pairs and right shoulder SNP pairs. Focusing on core-shoulder pairs, 8 of the 9 associations decreased between sampling years (Figure 5C; sign test *p* = 0.04, one-sided Wilcoxon signed-rank test, *p* = 0.068). This is expected, as a decrease in foreign allele frequencies in the shoulders but not in the core implies that recombination has dissipated the core-shoulder associations. In the right shoulder, 9 of the 10 associations decreased (sign test *p* = 0.022, one-sided Wilcoxon signed-rank test, *p* = 0.124), whereas in the left shoulder, associations increased between years in the six comparisons rather than decreasing. Thus, in addition to evidence of a decrease in *robusta* allele frequencies, there is evidence of LD falling between core and shoulder SNPs and between SNPs located on the right shoulder, as expected. Reasons for the observed asymmetry between the two shoulders, such as selection effects in the left shoulder, deserve further investigation.

## Discussion

We used ancestry-informative markers and KASP genotyping to analyze over 1,200 individuals of the native European sea squirt *Ciona intestinalis*. The aim was to monitor changes in the spatial distribution and frequency of adaptive alien alleles introgressed from the invasive species *C. robusta*. Our results indicate that *C. robusta* allele frequencies remain stable in the core of the introgression island on chromosome 5 over 20 generations while decreasing in the flanking regions, suggesting that introgressed alien tracts are eroding in frequency and length, and are concentrated in the center of the island.

### Multiplex KASP assay, a cost-effective method for rapid screening of native and nonnative *Ciona* species and their hybrids

The KASP genotyping approach was effective for species identification and detection of experimentally produced first-generation hybrids used as controls. However, no early generation hybrids were detected in the study populations.

As hybrids between *C. intestinalis* and *C. robusta* have been shown to be very rare in natural populations in previous years (Bouchemousse et al., 2016b,c), the absence of first-generation hybrids in our samples was expected. As our approach is based on the analysis of 11 SNPs from different chromosomes, it remains possible that successive backcrosses could produce a hybrid index close to the parents, although introgression tracts remain more numerous in these late-generation hybrids than in parental genotypes. Nonetheless, a hybrid index at 11 unlinked loci makes it possible to identify first and second-generation backcrosses with little ambiguity. We suggest that the use of our newly developed KASP method, based on the combination of both nuclear multiplex and mitochondrial single-locus assays, is an effective approach to identifying both native and introduced species as well as first-generation hybrids. A similar multiplex assay has also recently been successfully used to infer parental ancestries in admixed *Mytilus* mussels (Hammel et al., 2024).

Regarding the introduced *C. robusta* species, it is noteworthy that this species was found in only two locations, St-Vaast (site 10) and Arcachon (site 19), during the sampling made in 2021, despite active searches, as reported in the Supplementary Methods. Interestingly, among the four locations where *C. robusta* was sampled in 2012 (i.e., St-Vaast [site 10], Perros-Guirec [site 9], Brest [site 12], and Camaret [site 13]), it was found only in St-Vaast in 2021, where, moreover, it was rare. This observation is indicative of a decline in the introduced species. It is nonetheless too early to talk about a definitive decline, as Bouchemousse et al. (2016b) reported the reappearance of *C. robusta* in relatively high abundance in a site where it was thought to have disappeared (Nydam & Harrison, 2011). However, we can hypothesize that the introgressed *C. intestinalis* has now acquired the ability to outcompete *C. robusta* in an environment to which the latter was previously better adapted. Indeed, hybridization gives the native species access to a new gene pool; some of these genes may give the native species an advantage in habitats that are new to it in its natural range, as in the case of some harbor environments. With its preexisting advantages as the native species but now adapted to a new human-altered environment, *C. intestinalis* may take precedence over *C. robusta* in a habitat that initially benefited the latter for its stepping-stone worldwide invasion.

### Stable spatial distribution and frequency of adaptive alien alleles at the core of the introgression island

The KASP method allowed us to monitor introgressed alien alleles along chromosome 5. Targeting SNP 15 at the core of the introgression island, introgressed *C. robusta* alleles were detected in 13 out of 14 populations sampled in 2012 and 15 out of 20 in 2021, indicating that this introgression is widespread in the contact zone. Our 2021 sampling shows that the spatial distribution remains roughly the same and allowed us to detect three new introgressed populations not previously sampled, north and south of La Rochelle (site 18). Furthermore, as our approach enabled us to screen more individuals than previously done with RAD-sequencing by Le Moan et al. (2021), we were able to detect introgressed alleles in two populations, St-Vaast (site 6) and Roscoff (site 10), where they were previously undetected.

In both 2012 and 2021, the introgression appears to have a mosaic distribution in the species contact zone (Le Moan et al., 2021). Three regions stand out: Southern England, Western Brittany, and around the Gironde estuary, where *C. robusta* introgressed alleles are much more abundant. Furthermore, this mosaic distribution is consistent with that reported for neutral markers in populations of *C. intestinalis* (Hudson et al., 2016; Le Moan et al., 2021). It could, therefore, suggest that the initial introgression spread rapidly to the other regions thanks to shipping activities connecting these areas even over long distances (Touchard et al., 2022). However, a second key observation in our study is that both the spatial distribution and the frequency of *C. robusta* alleles at the core of introgression have remained stable over time, including in populations where the alien alleles were present but rare in 2012 (e.g., St-Vaast-La-Hougue). Adaptive alleles can spread rapidly in a heterogeneous environment through successive selective sweeps in the habitat where they are positively selected, as observed for example, in killifish, where pollution-resistant alleles spread from port to port (Lee & Coop, 2017). To explain the stability of low versus high frequencies of alien alleles at the population level, we must, therefore, consider the alternative hypothesis that the observed mosaic structure is driven by habitat heterogeneity rather than by the anthropogenic connectivity network of shipping. Under this hypothesis, populations where *C. robusta* allele frequencies are low and remain low in 2021 should have environmental components that counter-select alien alleles (as is often the case with resistance/cost trade-offs, e.g., Lenormand et al., 1999).

The peak of *C. robusta* allele frequency at SNP 15 located at the core of the introgression island is consistent with previous studies (Le Moan et al., 2021; Fraïsse et al., 2022). With only a single sampling time, these previous studies were left with the competing alternative hypotheses of (a) global positive selection with the adaptive allele on its way to fixation or (b) a kind of balancing selection, including local adaptation, that maintains introgressed alien alleles at equilibrium frequencies within populations. Our overall observation that alien allele frequencies at the core SNP 15 did not increase between the two periods (~20 generations for this species) supports the second hypothesis.

Local adaptation is a probable scenario based on our findings. Fraïsse et al. (2022) linked the core region to a tandem repeat of a cytochrome P450 gene (CYP2U1), commonly involved in detoxification processes. The function of this candidate gene, along with the stability of alien alleles, indicates that the introgression has now reached an equilibrium between migration and selection. It remains unclear what advantage this introgressed tandem repeat allele provides to explain its persistence. Looking at other cases of human-mediated adaptive introgression similar to ours (e.g., pollution resistance in fish (Oziolor et al., 2019), insecticide resistance in moths (Valencia-Montoya et al., 2020) and mosquitoes (Norris et al., 2015), or herbicide resistance and flowering time in maize (Le Corre et al., 2020)), we can expect that the selective advantage is associated with the specificity of the port environment (e.g., pollution, enclosure, substrate, …) where introgressed populations are found. A coupling of genotyping and environmental data at fine scales (within and between marinas) with functional studies will be needed to investigate this selective advantage in future work.

### Erosion of introgression tracts in the flanking regions surrounding the adaptive core

In the flanking regions, on either side of the introgression core (island shoulders), we observed a decrease in introgressed allele frequencies between the two studied time periods. In addition, we observed decreased pairwise linkage disequilibria between the core and the shoulders, as well as within one shoulder. This is likely explained by recombination breaking associations between linked loci after genetic hitchhiking and thus reducing the introgression tract lengths as alien alleles are getting eliminated by purging (Racimo et al., 2015) or gene flow (Bierne, 2010; Sakamoto & Innan, 2019).

The first explanation is that introgressed alleles that hitchhiked with the adaptive alien allele carry deleterious mutations in the native genetic background. Given the strong divergence between the two *Ciona* species, we expect most of the alien genome to be incompatible with the native genetic background and to be selected against when introgressed (Barton & Bengtsson, 1986; Dagilis & Matute, 2023). Even mildly advantageous alleles can easily cross species barriers with little delay, recombination allowing them to escape their association with genetic barriers (Barton, 1979). The delay depends on the selection coefficient and recombination rate, as well as the fitness effect of the mutation in hybrid genomes before it is freed from genetic barriers and can evolve adaptively in the recipient genome (Pialek & Barton, 1997). In low recombination regions and/or divergent parental genomes, an advantageous allele must escape a very dense background of counter-selected alleles linked to it on the genetic map. As in Peck’s “ruby in the rubbish” model (Peck, 1994), a beneficial mutation arrives in a genetic background where mildly deleterious alleles are dense. We expect recombination in introgression tracts to uncouple this negative selection during the selective sweep, making the hitchhiking footprint narrower than in a standard selective sweep model in which the shoulders are assumed neutral (Uecker et al., 2015). This expectation is in line with the Dagilis and Matute (2023) model that suggests short haplotypes are more likely to be introgressed than long haplotypes in divergent species where reproductive incompatibilities have evolved. Once the beneficial allele has fixed or stabilized in frequency, we can expect negative selection to continue purging alien ancestry in the shoulder of an adaptive introgression sweep, and this should be all the stronger as the divergence between species is greater.

The alternative, which is not mutually exclusive, explanation is that we are witnessing the second phase of a local sweep model (Bierne, 2010; Sakamoto & Innan, 2019). In this model, the sweep shoulders are assumed to be neutral. During the first phase (local hitchhiking), the locally adapted mutation increases in the population where it is beneficial, bringing with it flanking hitchhiker mutations. This results in the standard peak associated with a selective sweep, similar to the one we observe here in introgressed *C. intestinalis* populations. Local hitchhiking is fast and is followed by a second phase (interbackground introgression in the shoulders) that lasts for longer. Migration between habitats brings standing variation from unswept populations in the sweep shoulders. This model, therefore, predicts alien ancestry to be eliminated in the shoulder of the introgression island in populations where alien genes are favored at the core.

We also noticed a rebound of introgression at the right end of the right shoulder, at SNPs 20 and 21, notably in English populations, that was already observed by Le Moan et al. (2021). A focus on this particular genomic region shows the presence of another cytochrome P450 gene (CYP1F1) at the position of SNP 20. Genes of the CYP450 family often act synergistically on cellular detoxification, and clusters of introgression peaks on the same chromosome arm have been found to be associated with the CYP450 family in flies (Svedberg et al., 2021). However, we observed that *C. robusta* allele frequencies have decreased in 2021 at these two SNPs, similarly to other shoulder SNPs, so we could also propose that this introgression rebound around SNPs 20 and 21 would rather be due to chance association during the selective sweep in finite populations, or to negative selection being stronger in the chromosomal region of SNP 19.

Temporal monitoring of selective processes in natural populations is difficult and rarely documented, but doing so can offer insights into how it shapes introgression patterns. Here, our diachronic sampling provides evidence of the upholding of alien introgressed alleles in a specific genomic region in some native populations after an event of adaptive introgression. Meanwhile, hitchhiking introgressed alleles in the surrounding segments are being slowly eliminated by negative selection or flow of native genes, or most likely by their joint action.

## Supplementary Material

qrae016_suppl_Supplementary_Tables_S1-S6_Figures_S1-S4

## Data Availability

The multi-locus genotype table is provided in Zenodo. https://doi.org/10.5281/zenodo.8367591
