# Supplementary material for: Adaptive alien genes are maintained amid a vanishing introgression footprint in a sea squirt
Source: Evol Lett. 2024 Apr 27;8(4):600–9. doi: 10.1093/evlett/qrae016 (PMC11291672; doi:10.1093/evlett/qrae016)
Supplement: qrae016_suppl_Supplementary_Tables_S1-S6_Figures_S1-S4 [file qrae016_suppl_supplementary_tables_s1-s6_figures_s1-s4.pdf]

Supplementary Material

Adaptive alien genes are maintained amid a vanishing introgression footprint in a sea-squirt

Fanny Touchard, Frédérique Cerqueira, Nicolas Bierne and Frédérique Viard

Supplementary Methods

Detailed information regarding the material (samples and DNAs) and the molecular methods (DNA extractions, testing and genotyping procedure) used are provided below.

Sampling

Ciona intestinalis and *C. robusta* adult individuals were sampled in 22 ports from the North Sea to the Western Mediterranean Sea during late summer 2021, except for one locality (Dunkerque, site 5, Fig. 1 in the main text) sampled during summer 2022, totalizing 775 individuals (Fig. 1, Table S1 in Supplemental Material) (time period referred as 2021 hereafter). More specifically, *C. intestinalis* was sampled in 20 ports from the North Sea to the Bay of Biscay totalizing 666 individuals, and *C. robusta* was sampled in four locations totalizing 109 individuals. This latter species was easily sampled in two locations nearby the city Sète, in the Western Mediterranean Sea (104 individuals; sites 21 & 22 in Fig.1) but, although both species were actively looked for in the field along the other coasts of France, only five *C. robusta* individuals could be collected and in only two of the ports sampled in 2021, one in the North Sea and one in the Bay of Biscay (St-Vaast, site 6 and Arcachon, site 19; Fig.1). The two species were identified in the field on the basis of morphological criteria that are solid and easy to use (Sato et al., 2012). Note that there was almost perfect agreement between morphological identification and mitochondrial typing (see Results in the main text), a result consistent with previous surveys and studies (e.g., Bouchemousse et al. 2016a: only 1% discordance out of 3048 individuals). In our study, only one individual identified as *C. robusta* in the field was later confirmed as *C. intestinalis* with the mitochondrial SNP (see results in the main text). For each individual, a piece of branchial basket was collected and stored in RNAlater (samples from France) or absolute ethanol (samples from UK) for further molecular work.

For temporal analyses, we included DNAs of 439 *C. intestinalis* individuals, sampled in 2011, 2012 and 2014 (time period referred as 2012 in the main text). Most of these individuals have been analysed in the previous population genomics study that first revealed the introgression island (Le Moan et al. 2021). Additional ones from the same DNA collection were also included to reach ca. 30 individuals, as for the 2021 sampling.

The individuals analyzed in 2012 and 2021 were collected along the same pontoons (and by the same people) for all the marinas sampled twice in France, with two exceptions at Aber Wrac'h (site 11 in Fig. 1) and St-Vaast La Hougue (site 6 in Fig.1). At Aber Wrac'h, *Ciona* spp. were extremely rare and the six individuals finally found were sampled from different

pontoons throughout the marina. St-Vaast-La Hougue was sampled in 2012 by a colleague who did not inform us of the exact sampling location. Thus, in 2021, we sampled two pontoons that were randomly selected (in each marina, the individuals were sampled along two pontoons separated by another (unsampled) pontoon). In England, the populations were sampled both in 2012 and 2021 by the same person and using the same approach.

Samples outside the contact zone were collected in Coquimbo (Chile; site 24) for *C. robusta* and in Nahant (USA; site 23), Tromso (Norway; site 25) and Gullmar Fjord (Sweden; site 26) for *C. intestinalis* (17 individuals for each location). For the F1 hybrids, one was identified by Bouchemousse et al. (2016b) in one natural population collected in 2012 and eight were obtained from lab crosses using individuals collected in Brest (site 12) and Aber Wrac'h (site 11). These crosses were carried out similarly and at the same time as the parents-offsprings trios used in Fraïsse et al. (2022).

DNA extraction

When preserved in RNAlater, the tissues to be digested and extracted were first rinsed in PBS (1X) for one minute, then, for both tissues preserved in RNAlater and in absolute ethanol, around 20 mg was digested in 200 µL of lysis buffer (NucleoMag™ Tissue from Macherey-Nagel) and 25 µL of proteinase K at 56°C, 450 RPM for 3 hours in Eppendorf ThermoMixer® C. Extraction was done using Thermo Scientific™ KingFisher™ Flex System and followed the standard protocol of the NucleoMag™ Tissue kit except with 2x diluted magnetic beads. DNA concentration was measured with a Nanodrop spectrophotometer. DNA was normalised at 10 ng/µL using an NxP Span8 protocol.

KASP genotyping

SNP design and testing procedure

For KASP genotyping, one mitochondrial and 26 nuclear candidate SNPs, each with alleles diagnostic of the two species, were identified from sequences obtained in previous analyses (one from RAD-Seq data, Le Moan et al., 2021; five from transcriptome data, Bouchemousse et al., 2016c; 20 from whole genome sequences data, Fraïsse et al., 2022). The 27 candidate SNPs chosen for the genotyping step are detailed here: i) one SNP located on the mitochondrial genome to keep track of the maternal lineage, as previous studies showed that the hybridization between the two species is asymmetric, with F1 hybrids only arising from crosses with oocytes from *C. intest*inalis and sperm from *C. robusta* (Bouchemousse et al., 2016a; Malfant et al., 2018), (ii) 13 SNPs along chromosome 5, the focus of introgression (12 inside the introgression island and one outside (SNP "SB" in Fig. 2; from transcriptome data from Bouchemousse et al. (2016b)) and (iii) one SNP on each remaining chromosome (a

further 13 SNPs) to estimate a hybrid index for the remainder of the genome, as in Hammel et al. (2024). Using a combination of results from Fraïsse et al. (2022) with genome sequences and Le Moan et al. (2021) with ddRADseq but with larger sample sizes, we identified a SNP shared by the two datasets (SNP 15 in Fig. 2; SNP 38 radtag 877292-877271 in ddRADseq) that maps to the very core position of the introgression island. It is located 311 bp away from the end of the candidate cytochrome P450 tandem repeat. We thus expect a higher frequency of *C. robusta* alleles around this SNP in the receiving *C. intestinalis* populations. We used SNP 15 as a marker of introgression frequency, while the other 12 markers were used to study the extent of hitchhiking on the chromosome.

Primers for these 27 candidate SNPs were designed and produced by LGC Biosearch Technologies. The flanking sequences of each candidate SNP were carefully checked beforehand in order to limit the amount of polymorphism on each side of the targeted SNP and to facilitate the primer design. The GC content in primers averaged 42.8% with a minimum of 18.2% and a maximum of 65%.

Primers were first tested by genotyping 50 individuals for each SNP. Fourteen DNAs from previous studies with known genotypes (five *C. robusta*, two non-introgressed *C. intestinalis*, seven introgressed *C. intestinalis* and nine F1 hybrids) were used as positive controls. Thirty-six newly extracted DNAs were analyzed in the same run. For this testing procedure, one µL of assay mix (KASP-TF V4.0 2X Master Mix, 1X ; primers, 1µM ; HyClone™ HyPure water) and 0.5 µL of DNA were mixed in qPCR 384-well plates using Labcyte Echo525. End-point PCR was then performed according to the protocol provided in Table S2. The multiplex made of the 13 SNPs mentioned above was tested under the same protocol with all primers mixed together in equal proportions to maintain a target concentration of 1µM. For each DNA and assay, two fluorescence values were measured during the reading step, each associated to a given diagnostic allele. The fluorescence data were then analysed using the allele dosage or Variant Allele Fluorescent Fraction (VAFF) which is calculated as $\frac{f1}{f1+f2}$ with fi the fluorescence specific to the i allele (Hammel et al., 2024). The VAFF value indicates if, at a given SNP, an individual is homozygous (high or low value depending on the allele) or heterozygous (intermediate value). After the end-point PCR, the sum of the two fluorescences obtained was calculated and compared to the value obtained for the negative control to check for bad amplification. For genotyping accuracy, if the sum of fluorescences was equivalent to or lower than the negative control at a SNP for a majority of samples without any improvement after the recycling step (Table S2) or if the VAFF analysis gave unexpected results for the control samples, the SNP was discarded. To assess the accuracy of the multiplex, the VAFF calculated over all SNPs pooled together, used as a proxy for the hybrid index, was compared to the mean value of all VAFF obtained for each SNPs analysed separately. The correlation

between these values was calculated considering two genotyping test runs, the first one with the 50 selected individuals from both species mentioned above (R = 1; p < 2.2e-16 ; Fig. S1), and the second one with four positive controls from the first run as well as F1 hybrids (R = 0.99 ; p < 4.4e-11 ; Fig. A1 below).

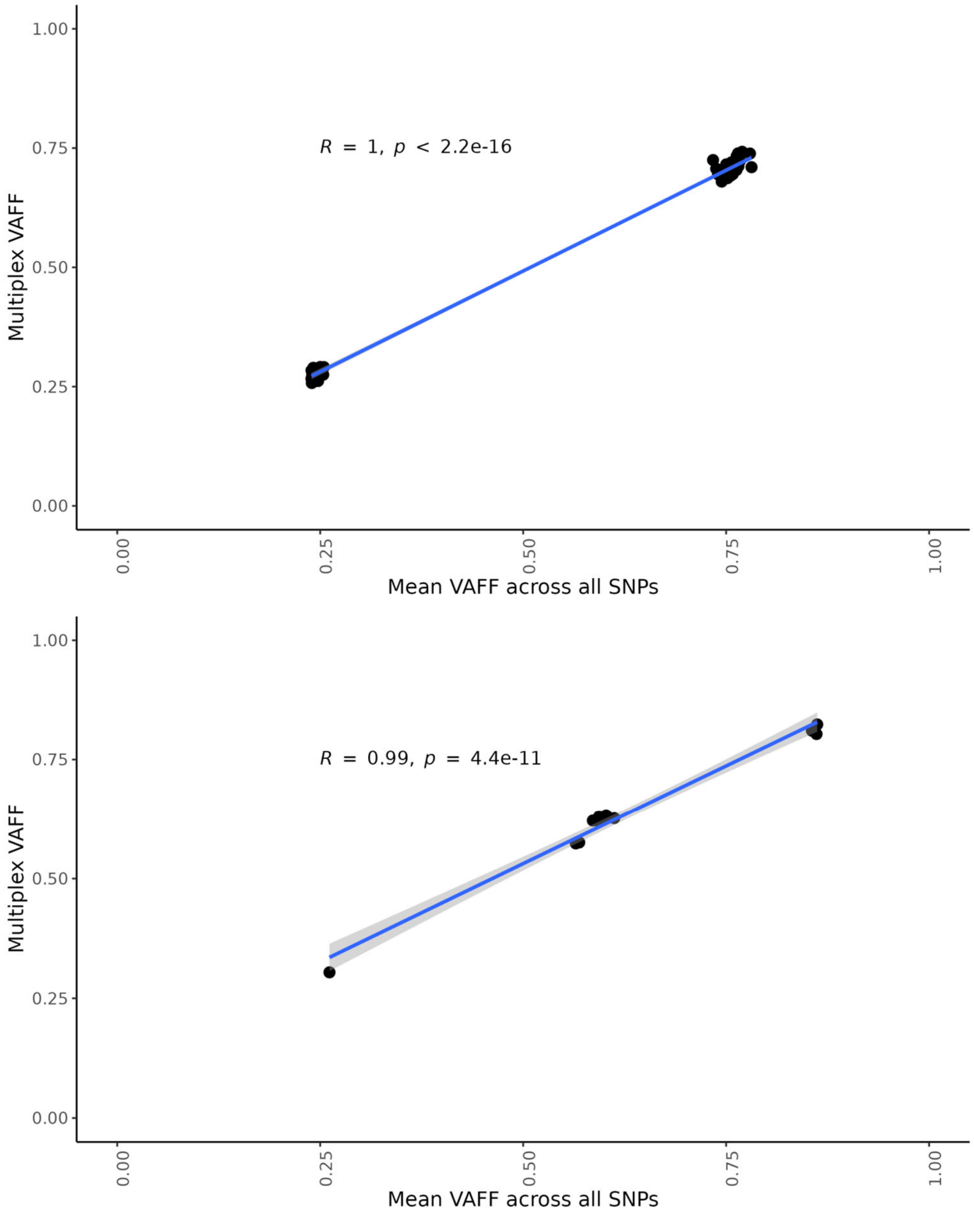

Fig. A1: Correlation of the multiplex VAFF and the mean VAFF calculated across all SNPs analyzed separately (simplex) with, for the top figure, 50 test individuals from both *C. robusta* and *C. intestinalis* and, for the bottom figure, with *C. robusta* and *C. intestinalis* controls chosen from the test individuals as well as F1-hybrid individuals (one found in natural populations in 2012 (Bouchemousse et al. 2016b) and 8 obtained from crosses in the lab produced at the same time than parents-offspring trios used in Fraïsse et al. (2022)).

Routine genotyping

Following the testing procedure, a total of 23 SNPs were kept, including 11 SNPs, one per chromosome (except chromosomes 8, 10 and 5), that were pooled in one multiplex (i.e. 1 assay) to compute an hybrid index, 11 distributed on chromosome 5 (10 in the introgression island including SNP15 at the core and 1 outside) to be analysed separately (i.e., 11 assays), and one located on the mitochondrial genome (Fig. 2 in the main text; Table S3). Of the four that were discarded, only one allele was amplified for two, and two did not amplify at all. Ultimately, a total of 13 assays (i.e. end-point PCRs) was made for each DNA. For the routine genotyping, only a few changes to the protocol described above were made. For three assays (SNP 20; SNP 21; mitochondrial marker), DMSO was indeed added to the assay mix (5% of the total volume). In addition, the recycling step used in the testing protocol did not improve the results and so, additional cycles were not done in subsequent reactions.

References:

Bouchemousse, S., Lévêque, L., Dubois, G., & Viard, F. (2016a). Co-occurrence and reproductive synchrony do not ensure hybridization between an alien tunicate and its interfertile native congener. *Evolutionary Ecology, 30*, 69-87. doi:10.1007/s10682-015-9788-1

Bouchemousse, S., Liautard-Haag, C., Bierne, N., & Viard, F. (2016b). Distinguishing contemporary hybridization from past introgression with postgenomic ancestry-informative SNPs in strongly differentiated Ciona species. *Molecular Ecology, 25*(21), 5527-5542. doi:10.1111/mec.13854

Fraïsse, C., Le Moan, A., Roux, C., Dubois, G., Daguin-Thiébaut, C., Gagnaire, P.-A., . . . Bierne, N. (2022). Introgression between highly divergent sea squirt genomes: an adaptive breakthrough? *Peer Community Journal, 2*, e54. doi:10.24072/pcjournal.172

Hammel, M., Touchard, F., Burioli, E. A., Paradis, L., Cerqueira, F., Chailler, E., ... & Bierne, N. (2024). Marine transmissible cancer navigates urbanised waters, threatening to spillover. Proceedings of the Royal Society B: Biological Sciences. https://doi.org/10.1101/2023.04.14.536605

Le Moan, A., Roby, C., Fraïsse, C., Daguin-Thiébaut, C., Bierne, N., & Viard, F. (2021). An introgression breakthrough left by an anthropogenic contact between two ascidians. *Molecular Ecology, 30*(24), 6718-6732. doi:https://doi.org/10.1111/mec.16189

Malfant, M., Darras, S., & Viard, F. (2018). Coupling molecular data and experimental crosses sheds light about species delineation: a case study with the genus *Ciona*. *Scientific Reports, 8*(1), 1480. doi:10.1038/s41598-018-19811-2

Sato, A., Satoh, N., & Bishop, J. D. (2012). Field identification of 'types' A and B of the ascidian Ciona intestinalis in a region of sympatry. *Marine Biology, 159*(7), 1611-1619. doi:10.1007/s00227-012-1898-5

Supplementary Results

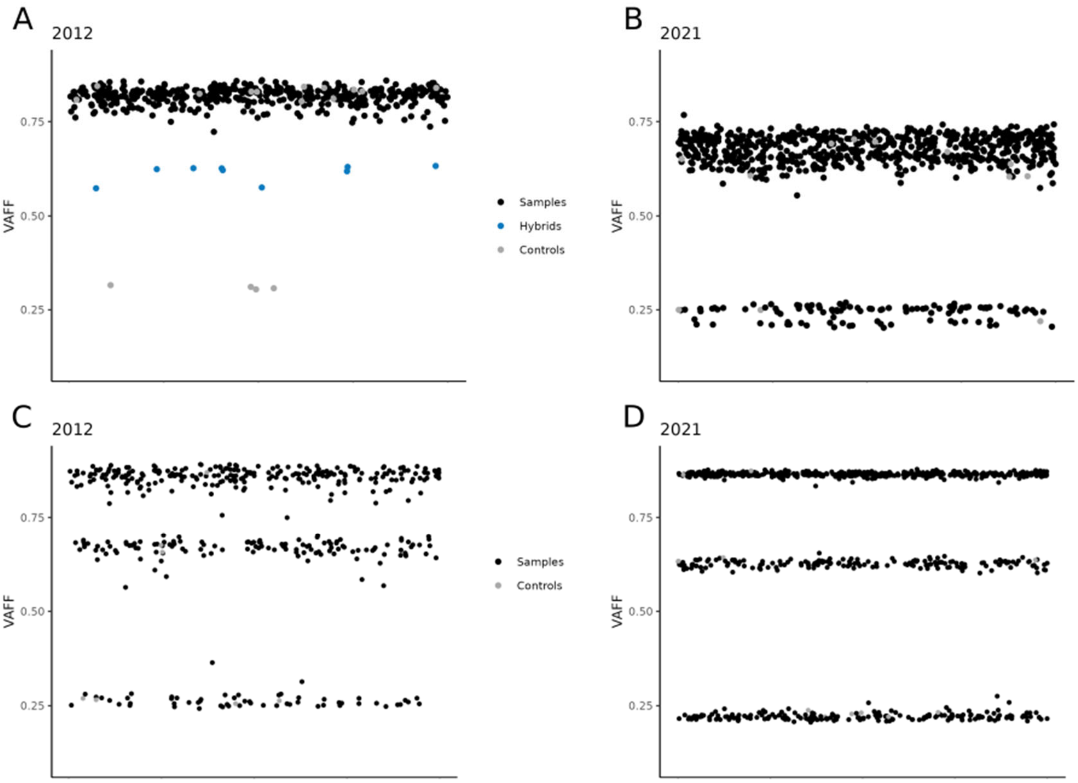

Fig. S1: Distribution of VAFF values for the multiplex for samples collected in 2012 (A) and 2021 (B) and SNP 15 at the core of the introgression island for samples from 2012 (C) and 2021 (D). Study individuals (black dots) are shown with controls, i.e. parental species, introgressed and non introgressed *Ciona intestinalis* (grey dots), and F1-hybrids (blue dots).

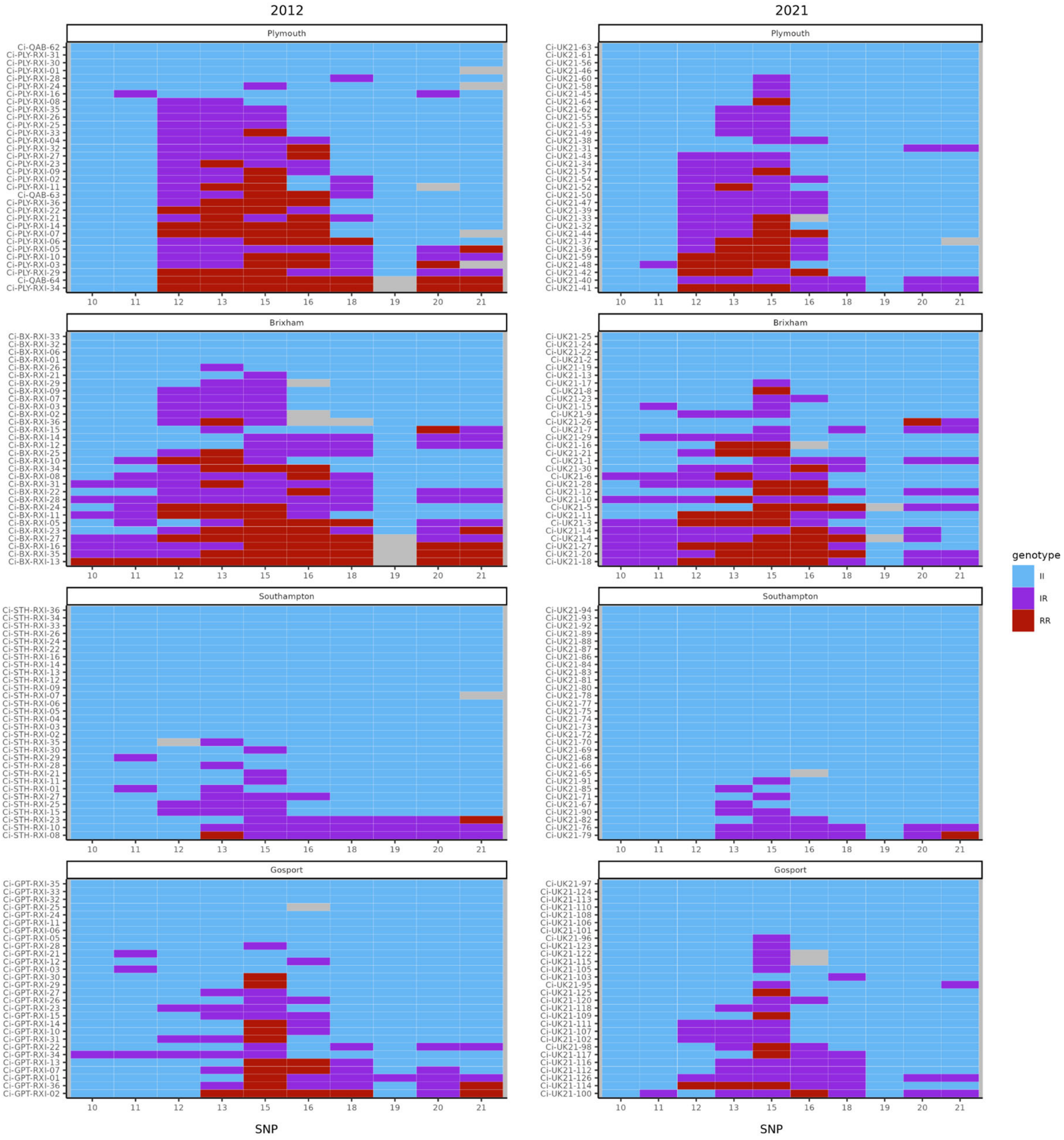
2012
2021
Plymouth
Brixham
Southampton
Gosport
10 11 12 13 15 16 18 19 20 21
SNP
genotype
II
IR
RR

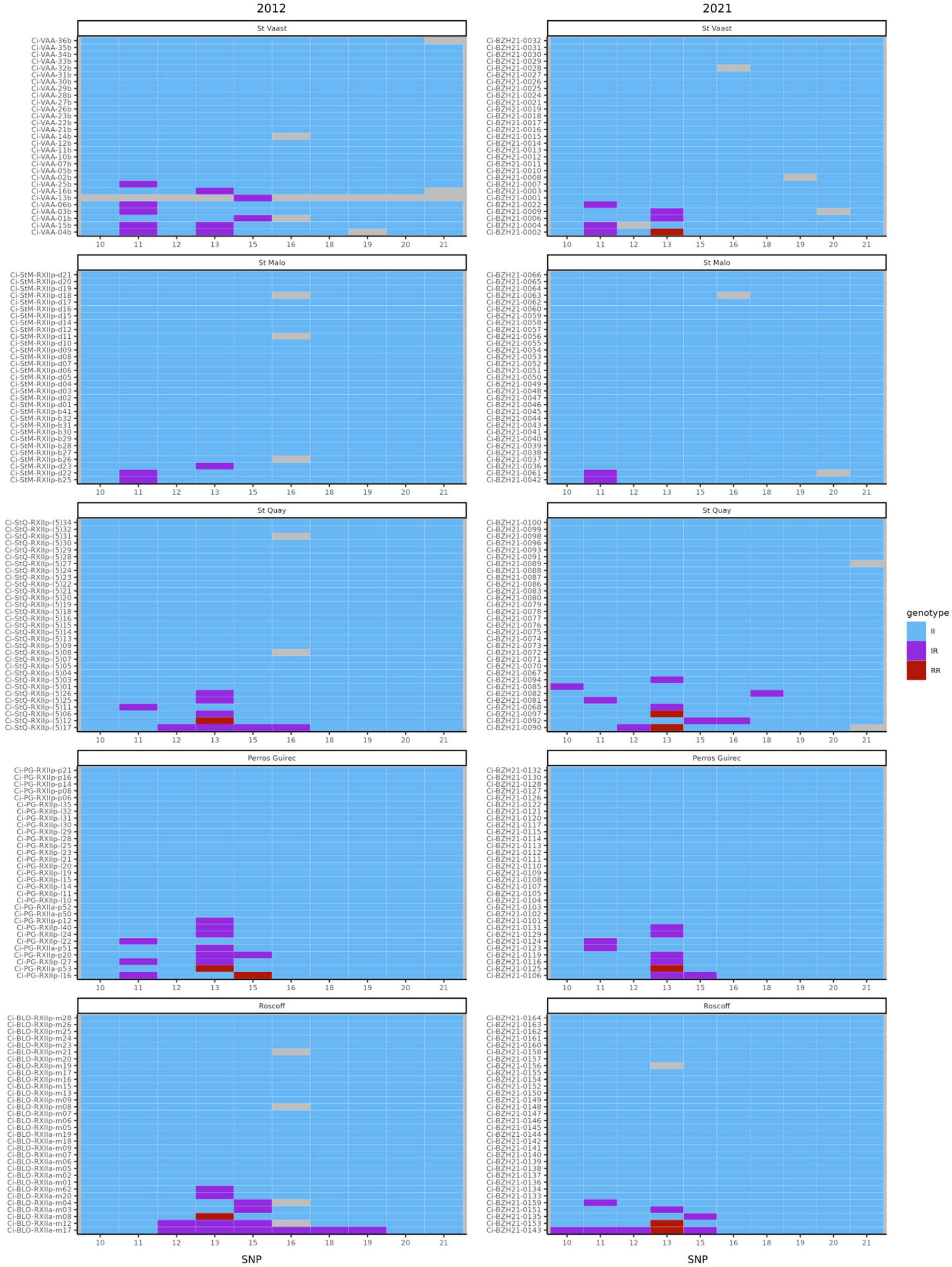

2012
2021
St Vaast
St Malo
St Quay
Perros Guirec
Roscoff
SNP
genotype
II
IR
RR

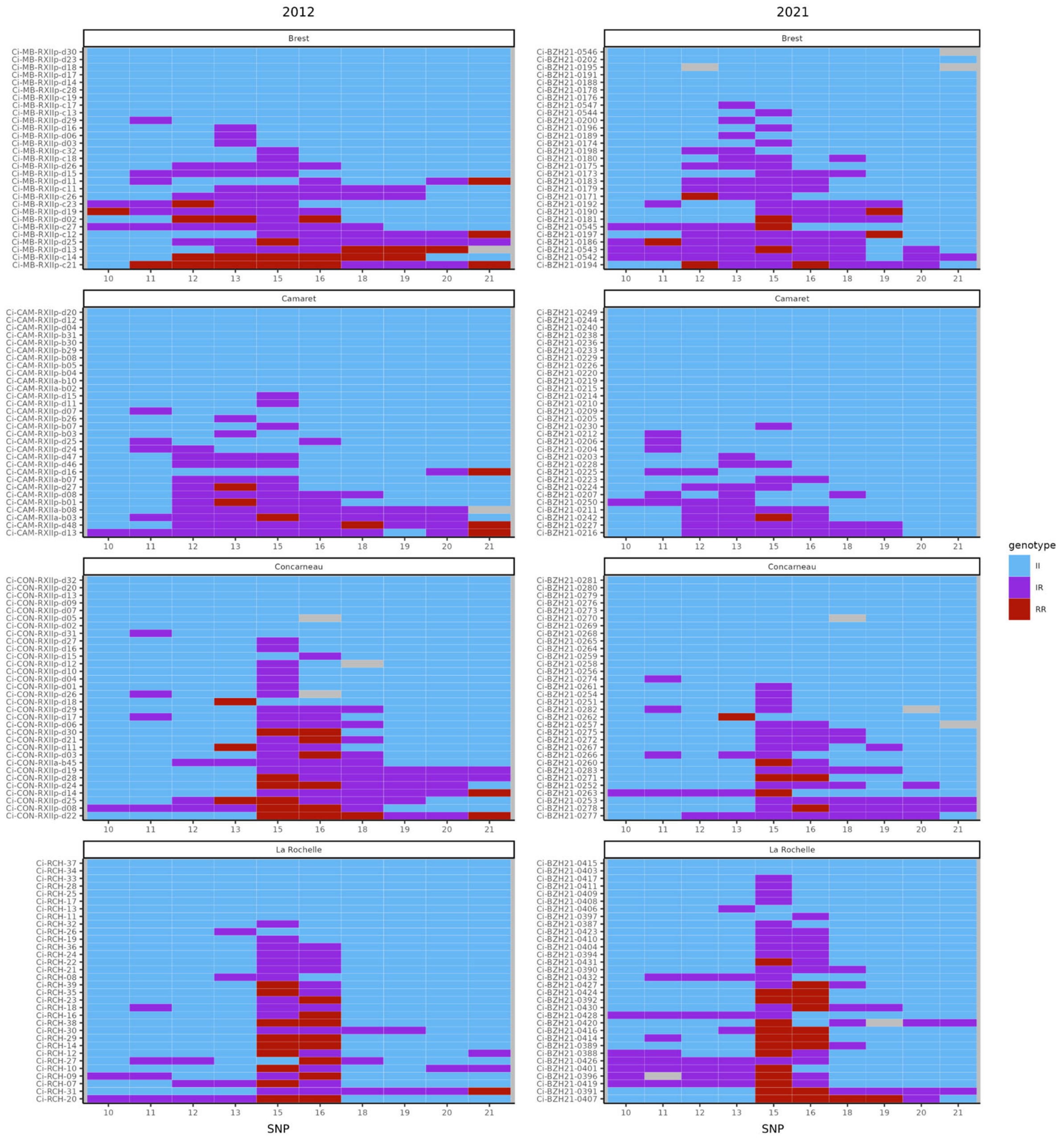

Fig. S2: Genotype of each individual for each SNP genotyped in the introgression region of chromosome 5.

For one given population, left and right panels correspond to 2012 and 2021 sampling, respectively. Blue is for *Ciona intestinalis* homozygotes, red for *C. robusta* homozygotes, purple for heterozygotes and grey is for missing genotypes.

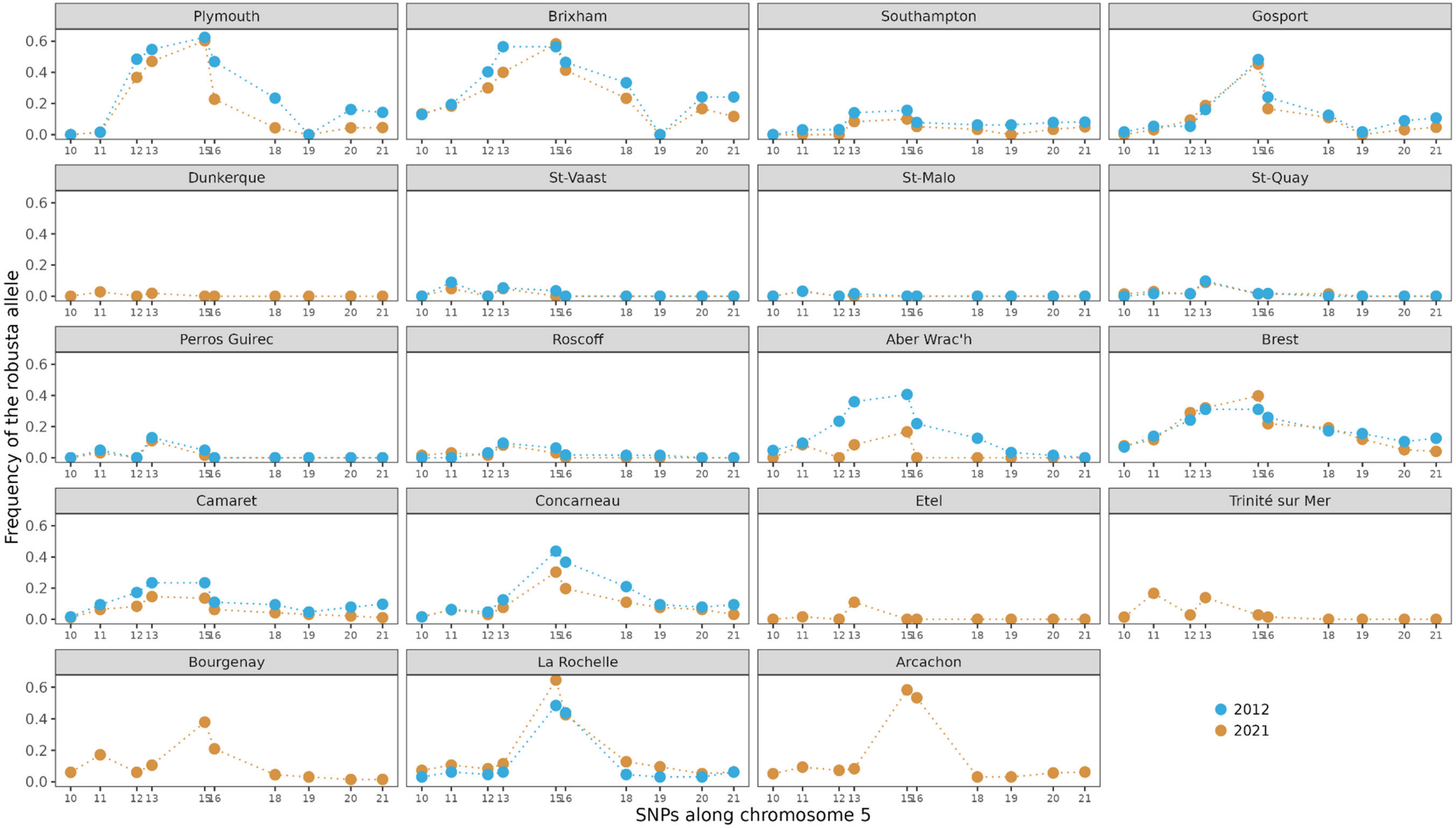

Fig. S3: Frequency of the introgressed *Ciona robusta* allele for each SNP analysed along chromosome 5, in each population and for the two sampling periods spanning 20 generations (2012 in orange and 2021 in blue). Note that the sampling size is balanced across populations (ca. 30 individuals), except at Aber Wrac'h where only 6 individuals could be sampled in 2021.

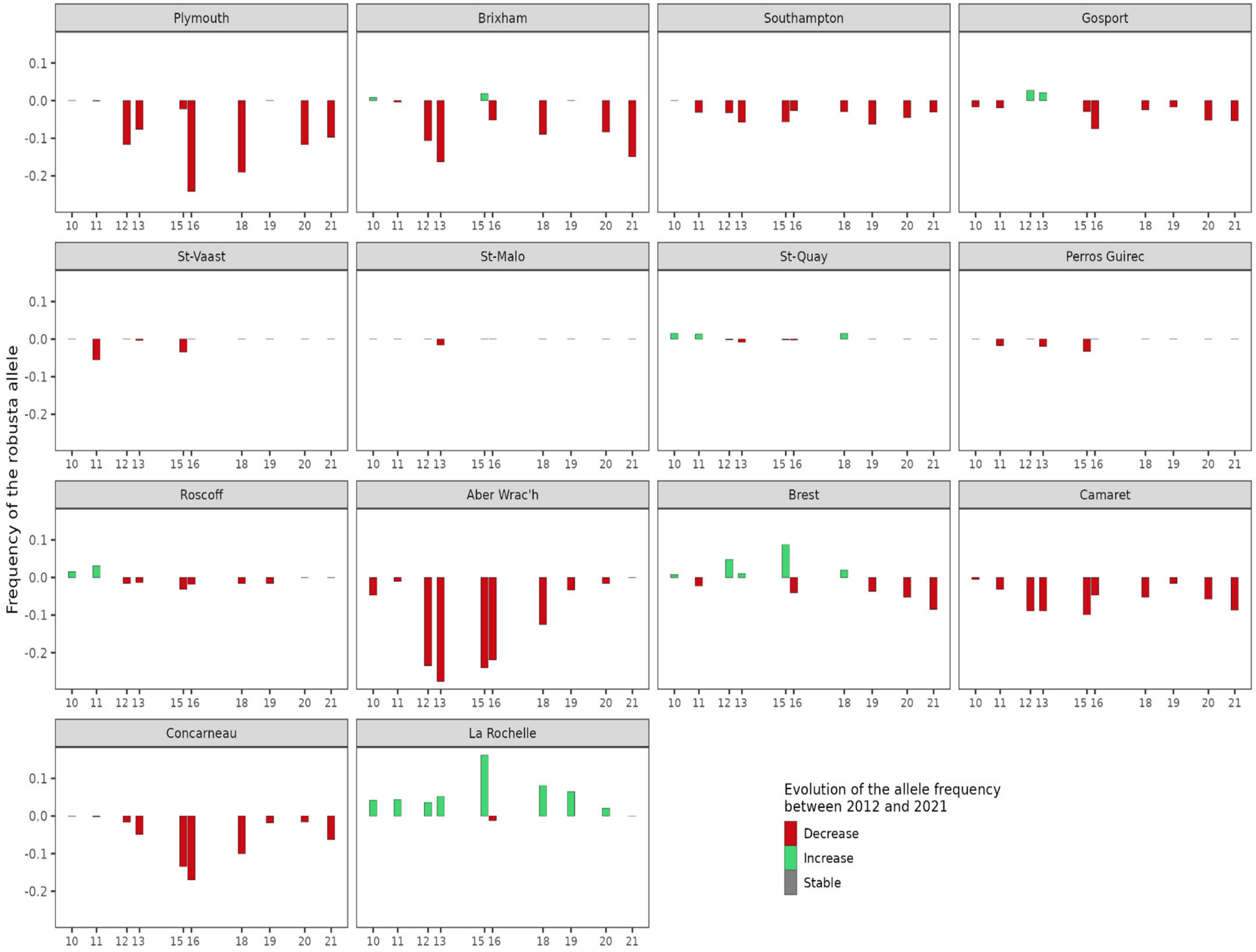

Fig. S4: Variation in frequency across 20 generations of the introgressed *C. robusta* allele for each SNP along chromosome 5, in each population

Table S1: Sampling number per localities, time period and species

The number (as shown in Figure 1 in the main text) and name of the sampling location are provided with the number of individuals collected for each time period and species. Ecoregions were named according to the classification of Spalding et al. (2007) [(1)].

Location number	Location name	Ecoregion	Number of individuals sampled in 2012		Number of individuals sampled in 2021	
			Ciona intestinalis	*Ciona robusta*	*Ciona intestinalis*	*Ciona robusta*
1	Plymouth	Celtic Seas	32	-	34	-
2	Brixham	Celtic Seas	32	-	30	-
3	Southampton	North Sea	32	-	30	-
4	Gosport	North Sea	30	-	32	-
5	Dunkerque	North Sea	-	-	54	-
6	St-Vaast	North Sea	32	-	32	2
7	St-Malo	Celtic Seas	32	-	32	-
8	St-Quay	Celtic Seas	31	-	34	-
9	Perros Guirec	Celtic Seas	31	-	32	-
10	Roscoff	Celtic Seas	32	-	32	-
11	Aber Wrac'h	Celtic Seas	32	-	6	-
12	Brest Moulin Blanc	Celtic Seas	29	-	39	-
13	Camaret	Celtic Seas	30	-	48	-
14	Concarneau	South European Atlantic Shelf	32	-	33	-
15	Etel	South European Atlantic Shelf	-	-	32	-
16	Trinité-sur-Mer	South European Atlantic Shelf	-	-	36	-
17	Bourgenay	South European Atlantic Shelf	-	-	33	-
18	La Rochelle	South European Atlantic Shelf	32	-	48	-
19	Arcachon	South European Atlantic Shelf	-	-	48	3
20	Hossegor	South European Atlantic Shelf	-	-	1	-
21	Etang de Thau	Western Mediterranean	-	-	-	32
22	Sète	Western Mediterranean	-	-	-	72
23	Nahant	Carolinian	17	-	-	-
24	Coquimbo	Central Chile	-	17	-	-
25	Tromsø	Northern Norway and Finnmark	17	-	-	-
26	Gullmar Fjord	North Sea	17	-	-	-

(1) Spalding, M. D., Fox, H. E., Allen, G. R., Davidson, N., FerdaÃ'A, Z. A., Finlayson, M. A. X., . . . Robertson, J. (2007). Marine Ecoregions of the World: A Bioregionalization of Coastal and Shelf Areas. *BioScience, 57*(7), 573-583. doi:10.1641/B570707

Table S2: End-point PCR protocol for KASP genotyping.

Step – Number of cycles	Program
Initialisation	15 min at 95°C
Amplification 1 – 10 cycles	20 s at 94°C then 1 min at 61°C
Amplification 2 – 29 cycles	20 s at 94°C then 1 min at 55°C
Read 1	1 min at 37°C then 1 s at 37°C
Recycling – 3 cycles	20 s at 94°C then 1 min at 55°C
Read 2	1 min at 37°C then 1 s at 37°C

Table S3: Flanking sequences and diagnostic alleles for the 23 SNPs kept for routine genotyping (see main text for details).

ID	Sequence	*C. robusta* allele	*C. intestinalis* allele	Position in genome
mitochondrial	ACTAGRGTTGATATAGCTATTTTTTCTTTRCATTTAGCTRGRGTTTCTA<br>GTATTTTAAGATCAGTTAATTTYTTAGTTACYTTATTTAATATAAARAATA<br>AAAGAAARTCWATAAGTAAYTTAAGTTTATTTTGTTGATCTTTAATTGTT<br>ACWACTATTYTMCTAGTAYTATCWCTTCCWGTTTTAGCTGCWGCWAT<br>TACNATATTATTATTTGAYCGWAATTTTAATACTACKTTYTTTGATCCKA<br>AYRGRAGAAGRGATCCWATTTTATATCAACATTTATTTTGATTTTTYAG<br>ACATCCAGAAGTTTATATTTTRATYYTACCWAGATTTAGAATAATTAGT<br>CATGTAATTGTYTTTTAYTCYAGAAAAGATAATATYTTTAGGTATTATAG<br>AATRGTTTGAGCWATRAGRGGWATTAGRTTTCTTAGRTTTYTAGTRTG[<br>A/G]GCTCATCATATATTYAGTGTARGWATAGATGTTGATTCTCGAGCTT<br>ATTTTACTTCWGCTACNATAATTATTGCWGTTCCTACWAGAATTAARG<br>TRTTTTCMTGAGTTTCAACWTTATTAAGRGCTAAWATTYAYTGARGATT<br>ACCWCTWTTATGAGCWTATRGATTTTTATTYTTRTTYACTATTAGARGR<br>TTAACTAGAATTRTTTTAGCTAATTGTAGTTTAGATCTTGTYCTYCATGA<br>TACWTATTATGTRGTTGCTCATTTTCATTAT	T	A	
chr1	GGTGAAGGGGGAATAAGYCTAACTGCACAGAAGTGCCCAATATTCCA<br>AG[C/G]CCATGGCACATCAGACCCCATGYTRCCRTTCCAGTTTGGSCA<br>GATGAGYAACATGCTGTTACAGAATGCAAGRAAGGATCTNAATCTHAC<br>CACTGAGTTTAAACCTTATCAAGGCATGGGRCACCAGTCATGTGAYG	C	G	1543504
chr2	GTGTCTTGGGTATCAAGGTTAAAATCATGCTTCCTTGGGAYCCACAAG<br>GTAAAATCGGACCCAAGAAACCTCTGCCTGACAATGTRAACATTGTTG<br>AACCTAAAGATGAAGAAGCCATCACTGGGCCAAGGAGTGARTCCAAG<br>GTTACCAAGCCRATGCCAGATCCCATGCCTGCAGC[T/C]CCACNGCAA<br>GCAGCTATGCCTCCMATGCAGCAGGTYCCACCCCAGCAAACCAWGC<br>CACCTATG	T	C	2666317
chr3	ctttaaaatatacagtttaaaactttmccaCCGCTCAAAAAAACATTGGAGTGCTTTTT<br>CCATTyAATAGCCGCGAACAAACGAkTGATTATGACTCGACAATAGAAA<br>ACGCGAAAAACGCrACCAAAACGATTTyGGCGTCACGGTTGA[G/A]CGA<br>CGTCACGTGTGTTGACTCAGCATTTCCAyAGCAAATCTTTCGAGAAAC<br>GAwGTCGATACATTAATAGTTACAATGTGTTTGTGTGTTGCaatgtgtgtgttt<br>gtgtttgtgtgtCTGTTCTCTCkTGTACGGtcatgtrtttgtttt	G	A	7030902
chr4	YATATGTAAGTTTCTATCAAAATGTCAGAA[C/T]AAAGATGGTGGTTTTG<br>GTGGTGGCCCTGGTCANNNNNNTCACCTTGCRCCCACTTATGCTGCA<br>ATTAAYTGTCTTTGCTCAATYGNAACCAARGARGCTTACTCYGTCATAA<br>AY	C	T	497855
chr5_SB	GTACAACCATTTGGTGTGGTGAATAATACATTTGGTCGAGCATTAGATT<br>GTGAAGGTTGGTTYACTATTGGWATATGGACGGGCYTGATTGTCACTT<br>TATTATTGGTAACAATTCTTACTCTTGGACT[A/C]TGCATGATTGCACAA<br>ATCACTACAATGGACAGATTTGATGATCCRAAGGGAAAACAACTTTCC<br>ATACCYCAACAAGAS	C	A	259572

chr6	taaatagacACCATTGTTGAATCATAGTTCTTTAATTGTGTCCTAACAaatgtt<br>attgtgatgtcatttatCkTGTATAACAGCAACTGTTGtgtgrattmtttttatygtcttaatattta<br>agttcaTAATGAATTGAATAACTGAtc[A/G]attgttacatcataawcatGAATTGTTT<br>ACAGGAGATTAAGCATTGTTACCTCACACTACTTGATCCCTAGTTAGrG<br>GAAACCCCCACCATAAATAATkGCATGAACttttatcccatcttatcatttataattagtttt<br>aaacattttt	A	G	4367328
chr7	ATGAGTTCTGCCAACAATGAAAACAAAGCTCC[T/C]GAGAAAGCTTCAA<br>CATCTGGTCGTGCWAMTGCTAGYTCHGCCAAAGATAATGCAAGTGAC<br>ACTTGGTCACTTAAAAACTTTGAYATTGGCAAACCAYTRGGACGAGGA<br>AAGTTTGGCAGCGTGTACCTTGCTAGAGARAARAAGAGCAAGTTTATC<br>GTTGCACTGAAAGTGCTGTTTAAATCRCAACTYATGAMRARTAATGTG<br>GARCATCAATTRCGAAGAGAGATTGAAATTCAGTCTCATTTRCGTCATC<br>CACACATTCTGCGACTTTACGGTTACTTTCACGAKGAGACGAGAGTGT<br>ATYTGATCYTGGARTATGCATCTCGTGGGGAAATGTACAARGAGCTGC<br>AGAAGCAGGGCAARTTTACAGMGGAGMTGTCCGCYACGTACATAGCY<br>GAGCTMGCAGATGCRCTCAACTAYTGCCACAGCAAACARGTCATTCAT<br>CGTGACATCAAACCKGARAACTTRTTGATGGGTCTTCGRGGGGARTTR<br>AAGATTGCTGATTTTGGTTGGTCTGTGCATGCTCCGTCYTCTAAACGC<br>CAAACYCTTTGTGGTACRCTTGATTACCTCCCRCCAGAGATGATTGAA<br>GCAAAAGATCATGAYGCTAAYGTYGAYCTGTGGACACTTGGSATTCTA<br>TGCTATGAGTTTCTTGTTGGCAAACCACCYTTTGAAACAAAAAGCACAC<br>AAGAAACATACCTHAGAATYACATCACTGAAATATKCATTCCCTCCCCA<br>TGTATCAGAGGGAGCWCGTGATCTTATTCGTCGCCTSTTAAAGTTGGA<br>ACCRCGACAYCGWCTTMCACTRGAAAGTGTRATGGCTCACCCCYGGA<br>TCARAGCCMATGC	T	C	3909404
chr9	AGTTCAATATAGAACAATATCGACAAACATGCACAGACGGGRAAAATC<br>AATASCAGTCTTTGTCGATTAAGTTAATCTCCGCTCTTAATATCCTGTC<br>AAASCGTGTACTTGTTATCCTTTGCTATTKCGCTGTGTTATAATTTAGS<br>GTCA[T/C]CGTTATAKAGCTKRAGGTACGCCRRGTTTATACAACATCGC<br>ACKTTTCCTTGTTAGTAGCTTTGAAARCGATTCTCMACACGTTTTTGGT<br>TCTTAATAATTGATAGCAGCACCATTAAGCCGAMCGTCTGGGCTCACT<br>GAAAGAAATTGT	T	C	5680810
chr11	GAAAMNRRAGGADTWCGARGAGATTATTTYATTTRTTAAGTCAAACAA<br>AWGCAAGAAATTAAAKRRNTTRATCWACCAAGTAAAHGATAWGTTTGA<br>AGYYAAATTTTCCTACCAAACATTRGTMAGCATATGTCTCATACACCAC<br>CAAAACCATGTRAAAAGAACWAKTCATGATAAAAGCACARATGYNWRY<br>RTRGAWAAATACTATTCYACMTTTTTACAAAGYAAAAKARATRRAAAAR<br>GCCAYRAAAACTCAGCAYTGRTGRAAATTGCAACYGAAWACRAYATTC<br>CACCTTTAYTRCTNGCTAGRTTRATTTTACATAGACWYCTWAAATTAAA<br>AGCASABVAAAGYRACCATRYKGRAACARVYVAAARAGTTAATYTYAAA<br>ACTGARGTYGCTCGACTKGTAAAAGATCCMTTTTTWATCRAAGACMAA<br>GKTTTMTCATGGGAAGTAMGRCAVTGCATTCTACATGATTTTGGTTAT<br>GGCCAAGTAACTGATTCAGTACGARGTCTYATTGGTTCAGAATATGAA<br>CACAAAMTAGTGACGHAYGTGAARVRRTTRAATATTCCATTTCARACY<br>GARGTTGATTTAAAGAAATTGGGATTTGATAAAACACCRGATATTAAAC<br>TTGAAATCCCATTTAAGTTRAAAVACCAAGTGGTKTGCTGGATTGAAAG<br>YAAAGCTTCTTTBGGCACTCCTGAAGAACATDTGTATTATGTGAATAAA<br>CAATACAA[C/T]AGCTACTGGAATCGATTTGGGCCAGGACTGGTGATCT	C	T	1623797

	ATTGGTTTGGTTTTGTTRATGARTTGGCGYWWGATATGCTCAAGAAGA<br>ATATCCTKGTTATGGATAGATTTCCAMTTCCTGCVGAAKTKTCYTTTTW<br>TAATCCTGCTTTWGTTGTWAAAGATGATAARCTATCA			
chr12	CCACTCACGACAGTACCAAAGCGTGTTTCCACTTCGAGGMCCAACGA<br>RCACGACCGTCTGCTGCACTATCACTCgacattatKacgtataYaTAGGCGCG<br>GGCGACCGCACGAAAGACGCAACAGAGCGAAATCTWSAGTTCTCGCT<br>GTR[A/G]AACCACAGMTTTGTTACATGCGCTACAACTTTAACGGCGATT<br>TTTACTGTTTAGTTTGCGTGTCYACAGTAGCGCTCGTGTTTCCTGTAAG<br>CGTCCGSCGTGGCTTGTTGCCGTTTCCCGACTRCATGGTCGTGTTGT<br>GTCGTCACGGCG	A	G	6173939
chr13	CGGYCGGCCKRTCGGTTAAATAATTCTCGATRTAATTTTCGGCGCATC<br>GGTTACGGCTWGGGGGACGTGGGAGTGTGTTATGTTTTGGTGTYGAY<br>TCTAAATCGGTACGAGGRGGCACTGGRGGGCAGTCRAGRTCATCGCT<br>GGTRATTC[G/T]TTTYAAGCGTAACGTGACGTCACCKCCCATGTCRCTC<br>GCATTCCACGgaaaactgaaaacaaaaaacaatatttaggcataaatttaaWttgtgaaaWa<br>attgAACTGTCGatgtatttatMtcttcgaatacagtagcGAATTCAAACA	G	T	2394321
chr14	GMGTCTAATTAAAAAATCGCTTCGTYTTTTTACAAATTGCRTTCAAGTG<br>RCRCAAGAAAGATCAAATATCccaaattcaWttaaataacttaaatacatTTCGAAC<br>ATTGTAACTGTATTGMGAGGACCACTGCRGCAAACCAGCGCGAT[G/A]<br>GGAATTGCAGCTCTTACRTGGCAGGTGTTTGAACAAGTTGCCACTAAA<br>CTTCTAGTTGTTCTCTCTCATSTCTTGGTTTATAATRAGCGAYTCGCCC<br>AAGTATCTGATACGGACYTCACCCAATCCTCTATTGYTTGCTTCTAYTC<br>TGTG	G	A	159786
chr5_10	ACCTCCACTAGATTTYAATGTCTAAGWAMTYSCATGTTTGTTAAGGTCA<br>AAGGAGTTTTCAAGYTTCATATCTCGATGTTTGGTRAAAGATCCAACRA<br>AAAGAGCRTCGYTAGCCGAGTTGCTTAACCATCCTTTCCTCACCAAYA<br>TCTC[G/A]TCMCCACAACCTCTTGTKAAGTTGATCGCTGAAGCAAAAGC<br>TGATGTCACAGAAGAAGTKGAAGAACCTGTGagaattattttgtYYRttttaaatYtg<br>ttGATTWCCAATGTGTTCTGGTTTTATGAAAATTTGAACCTAGTTAAGAC<br>T	G	A	738970
chr5_11	ATACAATCYCARCYTGTGAACAWTCTTACTCATGAGCAACTACGGGTT<br>GTGAATCATGATTTTCAAACTGGTCAGTTGATWCGAGTAATGGCTTAT<br>GCYGGTGCAGGAAAAACAAGCACRTTGATYGCTTGTGTTAAAGCTAAG<br>CCAAAC[A/C]AGACATTCCTATACACCTCATTTTGCAAGTAGGATTGTTT<br>TACAATGTCTATAAATCTTGTYACTRRAAgRttcagtttttttttctgttataatttMtgttac<br>tgtttttaacaataggCAACCATAAACAATTATTAGTCTGRTAARG	A	C	808581
chr5_12	RCARAWAATTGATATTAGTAACCACCGTTCAATCATTACCACCTMGGC<br>TTACWTTCAAATGCGTCATTTGCTTGTTGACGTGACCACAGACTGTCS<br>TCGTASGCRGCGCTTTGAATRATYACATTCGAAAATAATMCTTCGYTCT<br>TTGGA[T/G]AGAGGGCATGCATGTGAACSGAGGAGCCACCGGCCTCTT<br>CRCCAAATAKAGTAATTYKAGYWGGATCGCCGCCGAAMgcctgtaaaaaa<br>WtacaacWYtTAGRTAARCCTATTAAGGACCRCAAACACAgatataRgtttttaa<br>ataaWa	T	G	894811
chr5_13	WAACATGTATGAGCCTGGAGATGGTATACCCCCACATACYGAYAACA<br>CAAGATCYTTTGATGGTGTGTTATCMACYGTAAGCTTGGGTTCACATA<br>CTGTTATGAACTTTAGYAAAGAMGGTGCTGAAAGAATAGATGTGTGTG<br>TCGAGCC[G/A]cgtactttatttttatttactggtGAATCAAGATATGAGTGGAGACAY	G	A	930715

	GGCATCCAGCARAGAAAGTTTGATATTTTRGATCAGGGGRMAAAAATAACCACTCGSACCATTMGSTATTCYTTGACATTTCGAACAGTTGTAAAAGATGCY			
chr5_15	ACAGATAAAATCWTTCTAACATTTAAGTGAAAAAGTAATGGAGGTMCAAGTTATAGAAGTAACRAAAGRAAATCTTGAGCYATTATGGAGTAAAATCGTGRAAGAYGYGAAAAACTCCATTTTYATTGCACTAGATACRGAAATGAGCGG[A/T]TTAGGACCCCAAGCMCAGCTWATGAAGTCTGACTTGGASCAAAGGTATCARGGWATTTCTGCAGCAGCGTCWTCCCGTTCTATTATCTCTCTTGGACTTGCTTGTTTTGAGMCRTATTCTGGRAAAGAACCRCTGGCCTATAATGTAACT	A	T	1055311
chr5_16	CACAAATATACCCAAATTGCCAGATTAGCGTCAAGTCGGCGTAATAGCGACAGGAGGCTGCATGCTGGGTAYACAGACGAGAGTGCACCAGCATGGSAAGGGCATAGCGCGAAGAGAAGATTACGCAATAACTTAGAGTACATGTGTGCG[C/T]CGCATATATTTCRTASTGYGGGGYCATGTGGGATAYCTTTatcacctaaatcccatatttcttgatcgtgttttaaacacctgaAACCACTCCTTTAGAGTGGTGGGGctacagttaaaaaattccttaaatattctttgtttactactt	C	T	1078275
chr5_18	GCTCAAAATTAAGAGAAGGGATACTGCTTGAAGTACGGATTTGGGGCGCCTAGTAAAGAGGGTCagaattaaacataatacattgtttttcTAGCTAAAAMCGGACCAATAGTTTCAAAGGGTTAGGTTTAATATAGCATTACGAWATGG[A/C]GCGTGTTCTTAAATTAWCGTTTTCTTTGAAATTTGTACAAAATAGATTGAAATCCAATTGTGGTGTCGCGATATGACCAAAGCTTTAATATTATAACTGYCATGGCTTGTAATTTATGTCGCGGAACATAAAACCAGTATATAATGATTA	A	C	1221328
chr5_19	ARAAYGTAATTCAGCACATACTTMRAAACAGAGACAGCGTAATCATACATAACACATCAGTAGTTTGACCTTACATAACCYARCATATAACAAGACAGGAAGTGAATGTGGAAATTGCTATTTCTTCTTATTCAGCTGGACATRACACAG[T/C]TGCAGAAGGCCYATCTTAYCATAGCAATGCAATGTTATTGAATTggacaacaacaaaaWttgWaaagaTCAATGCYWCATAATTGccaRWtttattaaagMaYATGATATYCACAATATTGCATGGTTGACTAACYTATTACTGCTAYGC	T	C	1301011
chr5_20	gacgtaataagcaTGCsAwTGTGCAAGTCACAATGAATTTGTTGTCTGCATTGCGTCACAGTAAACCGATTGTTCGTGGAAAACkCGGTTAATGGTGTCAACAACyAACAyGrAGCrAATGTATCGCGAAACTTGTCTAGTGGTTGCGAk[T/C]GCGGAwGTTGGTCwACCTTGCTACTCGTGCGAAkGTAATTATCrGAATAATTyAGATTAACGCATCACGTTTAGTrTGTGTCGTCATAATGAArTCAyTGTCGGyTGCCAATCkkTATTTTrCATGTwACAACGAwTrrGCAGTTCTGTC	T	C	1400189
chr5_21	taaacaaacgAACCACGCGCCrAGATCTCACCACAGATAAGACGGTAATGGTTCGATGTGAGGTTGTCCAATCTCGCATTTTGCTTTGCATCGTCGCGCTGCTATGTCAACCGCTwAGAAAACATTCAAwTTACAAAAGTTTCTTTTTAC[A/T]ATTTCATAACGATCGACATAGCTTyATATTGCAGCAGTTGTGTATTGCTAATGCAAGyGmATTAACCACACACAACTGCAsTGTTATCTAAAATGTTTACCATCCTTTCTTGGCAGTTTAAACTCCCCTGCGAATAAATAAGATATCTGT	A	T	1475058

Table S4: Cut-offs values based on VAFF values to determine homozygotes for each allele and heterozygotes as well as pure *Ciona intestinalis*, pure *C. robusta* and hybrids for the multiplex. Heterozygotes and hybrids had values in between the two cut-offs indicated in the table.

	**2012**		**2021**	
**SNP**	**Cut-off homozygotes *C. intestinalis***	**Cut-off homozygotes *C. robusta***	**Cut-off homozygotes *C. intestinalis***	**Cut-off homozygotes *C. robusta***
mitochondrial	> 0.70	< 0.40	> 0.75	< 0.30
Chr5_SB	> 0.60	< 0.40	> 0.60	< 0.30
Chr5_10	> 0.70	< 0.40	> 0.60	< 0.30
Chr5_11	> 0.75	< 0.4	> 0.70	< 0.30
Chr5_12	> 0.70	< 0.40	> 0.55	< 0.30
Chr5_13	> 0.60	< 0.40	> 0.60	< 0.40
Chr5_15	> 0.71	< 0.40	> 0.80	< 0.30
Chr5_16	> 0.70	< 0.35	> 0.70	< 0.35
Chr5_18	> 0.70	< 0.40	> 0.70	< 0.40
Chr5_19	> 0.70	< 0.40	> 0.75	< 0.40
Chr5_20	> 0.80	< 0.50	> 0.70 (run 1)<br>> 0.82 (run 2)	< 0.40
Chr5_21	> 0.60	< 0.45	> 0.60	< 0.31
	**Cut-off pure *C. intestinalis***	**Cut-off pure *C. robusta***	**Cut-off pure *C. intestinalis***	**Cut-off pure *C. robusta***
Multiplex (11 SNPs)	> 0.70	< 0.40	> 0.50	< 0.30

Table S5: p-values associated with A) Fisher's exact test on genotype counts per population and over the whole dataset at SNP 15 (core of the introgression) ; q-values obtained after adjusting for multiple testing are also indicated for each population; and B) Fisher's exact test and one-sided (2012 values greater than 2021) Wilcoxon signed-rank test on allele frequencies, for examining temporal changes in frequency of the foreign *Ciona robusta* allele, in the shoulders of the introgression island, between 2012 and 2021. p-values below 5% are indicated in bold.

A) Test for temporal changes at SNP 15 (i.e., the core of the introgression island)

Site number	Site	p-value	q-value
1	Plymouth	0.857	0.932
2	Brixham	0.857	0.932
3	Southampton	0.427	0.695
4	Gosport	0.856	0.932
6	St-Vaast	0.232	0.602
7	St-Malo	NA	NA
8	St-Quay	1.000	1.00
9	Perros Guirec	0.360	0.682
10	Roscoff	0.679	0.931
11	Aber Wrac'h	0.126	0.482
12	Brest	0.367	0.682
13	Camaret	0.137	0.428
14	Concarneau	0.148	0.428
18	La Rochelle	**0.049**	0.428
Over the whole dataset		0.494	NA

B) Test for temporal changes for each SNP located in the shoulders of the introgression island

Locus	Fisher p-values	Wilcoxon p-values
chr5_10	0.655	0.639
chr5_11	0.627	0.093
chr5_12	0.058	0.099
chr5_13	**0.015**	**0.018**
chr5_16	**0.005**	**0.002**
chr5_18	**0.028**	**0.034**
chr5_19	0.680	0.091
chr5_20	**0.009**	**0.012**
chr5_21	**$6\ 10^{-4}$**	**0.011**
Across all loci	**$3.41\ 10^{-6}$**	**$1.91\ 10^{-6}$**

Table S6: r² values (mean value per population, with standard deviation) for linkage disequilibrium between each SNP for both time periods, in 2012 (top) and 2021 (bottom).

2012	SNP10	SNP11	SNP12	SNP13	SNP15	SNP16	SNP18	SNP19	SNP20
**SNP11**	0.331 ±0.191								
**SNP12**	0.182 ±0.096	0.117 ±0.137							
**SNP13**	0.071 ±0.057	0.051 ±0.08	0.39 ±0.242						
**SNP15**	0.063 ±0.073	0.073 ±0.099	0.38 ±0.305	0.188 ±0.192					
**SNP16**	0.096 ±0.072	0.083 ±0.087	0.352 ±0.374	0.193 ±0.165	0.48 ±0.209				
**SNP18**	0.083 ±0.133	0.07 ±0.145	0.145 ±0.156	0.122 ±0.091	0.28 ±0.134	0.518 ±0.29			
**SNP19**	0.009 ±0.011	0.012 ±0.011	0.115 ±0.177	0.078 ±0.079	0.223 ±0.148	0.388 ±0.379	0.67 ±0.339		
**SNP20**	0.082 ±0.118	0.045 ±0.051	0.044 ±0.063	0.048 ±0.045	0.15 ±0.077	0.25 ±0.204	0.475 ±0.227	0.502 ±0.297	
**SNP21**	0.095 ±0.138	0.048 ±0.068	0.044 ±0.07	0.054 ±0.069	0.121 ±0.085	0.22 ±0.213	0.413 ±0.241	0.343 ±0.315	0.765 ±0.09

2021	SNP10	SNP11	SNP12	SNP13	SNP15	SNP16	SNP18	SNP19	SNP20
**SNP11**	0.39 ±0.264								
**SNP12**	0.377 ±0.361	0.196 ±0.244							
**SNP13**	0.204 ±0.2	0.108 ±0.134	0.503 ±0.18						
**SNP15**	0.128 ±0.166	0.063 ±0.066	0.218 ±0.172	0.166 ±0.132					
**SNP16**	0.057 ±0.128	0.057 ±0.079	0.163 ±0.172	0.134 ±0.087	0.395 ±0.27				
**SNP18**	0.034 ±0.053	0.022 ±0.015	0.077 ±0.066	0.115 ±0.121	0.124 ±0.111	0.362 ±0.225			
**SNP19**	0.019 ±0.018	0.015 ±0.007	0.112 ±0.149	0.051 ±0.061	0.092 ±0.038	0.328 ±0.113	0.544 ±0.218		
**SNP20**	0.033 ±0.042	0.045 ±0.078	0.034 ±0.026	0.065 ±0.122	0.063 ±0.094	0.2 ±0.211	0.38 ±0.295	0.264 ±0.236	
**SNP21**	0.006 ±0.006	0.025 ±0.046	0.025 ±0.036	0.058 ±0.106	0.05 ±0.084	0.158 ±0.187	0.293 ±0.272	0.223 ±0.158	0.663 ±0.206
